# Pyrodiversity of boreal lake islands begets biodiversity of beetles, plants, and birds

**DOI:** 10.1002/eap.70218

**Published:** 2026-03-30

**Authors:** Aaron J. Bell, Stephen M. A. Paterson, Steven L. Van Wilgenburg, Colin P. Laroque, David A. Wardle, Iain D. Phillips

**Affiliations:** ^1^ Department of Biology University of Saskatchewan Saskatoon Saskatchewan Canada; ^2^ Troutreach Saskatchewan, Saskatchewan Wildlife Federation Moose Jaw Saskatchewan Canada; ^3^ Department of Environmental Science Saint Mary's University Halifax Nova Scotia Canada; ^4^ Environment and Climate Change Canada, Canadian Wildlife Service Saskatoon Saskatchewan Canada; ^5^ Mistik Askiwin Dendrochronology Lab University of Saskatchewan Saskatoon Saskatchewan Canada; ^6^ Department of Ecology, Environmental and Geoscience Umeå University Umeå Sweden; ^7^ Ecological and Habitat Assessment Services Water Security Agency Saskatoon Saskatchewan Canada

**Keywords:** fixed‐area sampling, habitat amount hypothesis, island biogeography, island disturbance, patch mosaic burning

## Abstract

Global fire regimes are changing, raising concerns about the ability of fire‐prone ecosystems to maintain biodiversity. We tested whether the pyrodiversity–biodiversity hypothesis (i.e., variation in postfire characteristics promotes biodiversity) or alternative hypotheses better explain patterns of biodiversity in a true island system. Using fixed‐area sampling plots in a chronosequence of 42 boreal lake islands spanning gradients in island area (1–350.4 ha), isolation (0.1–7.9 km from mainland), and fire history (1–231+ year since fire), we tested whether alpha and beta diversity of beetles, plants, and birds increased with spatial (within‐island variation in burn severity) and temporal (variation in time since fire among islands) pyrodiversity, respectively. Species richness of plants and birds increased with spatial pyrodiversity indicating that habitat heterogeneity from localized variation in burn severity supported more species in some groups. Beta diversity of all taxa increased with temporal pyrodiversity, highlighting the importance of conserving age‐class variation within the boreal patch mosaic. In contrast, the habitat amount hypothesis and island biogeography theory were weak predictors of species richness across all taxa, and island area and isolation did not consistently affect beta diversity among the islands. Our findings emphasize the importance of maintaining pyrodiversity in boreal landscapes to combat biodiversity loss in the age of “megafires” and suggest leveraging the fire refugia effects of large lakes within the region to conserve vital components of temporal pyrodiversity such as old‐growth forests.

## INTRODUCTION

Global temperatures in 2023 and 2024 were the hottest in recorded history (Wong, 2023) and gave rise to exceptionally active wildfire seasons in Canada (Owens, 2023), where the annual area burned far exceeded the 10‐year moving average in both years (CIFFC, 2023; Jain et al., 2024). In addition to these increases in wildfire being linked to climate warming and more frequent extreme fire weather events (Cunningham et al., 2024; Jain et al., 2024; Kirchmeier‐Young et al., 2019), they are also facilitated by fire suppression and land use changes that have influenced the distribution, build‐up, availability, and type of fuel (Campos‐Ruiz et al., 2018; Moritz et al., 2014; Sayedi et al., 2024). Increasing wildfire activity is also causing shifts in contemporary fire regimes towards longer fire seasons and a greater number of days annually suitable for fire spread, as well as increases in both annual area burned and the frequency of large (>200 ha) wildfires (Hanes et al., 2019). In the boreal forest, where habitat composition and configuration are heavily influenced by wildfire (Erni et al., 2017; Stralberg et al., 2018), such landscape‐level changes combined with anticipated increases in wildfire activity (Wang et al., 2017) have the potential to reduce biodiversity by decreasing habitat heterogeneity of the patch mosaic. Larger, more frequent, and uniformly severe wildfires in the boreal (Collins et al., 2025), for example, might reduce biodiversity by decreasing the proportion of old‐growth forests (i.e., reduced stand‐age heterogeneity, Erni et al., 2017) or by reducing spatial variability in postfire legacies and recovering vegetation (Johnstone et al., 2016).

Concurrent with ongoing changes in wildfire activity, there is growing interest in expanding protected area networks in the boreal forest due to its high ecological value and conservation potential (ECCC, 2023; Powers et al., 2016; Schindler & Lee, 2010). Although scientists have long debated how best to manage landscapes for sustaining biodiversity and have recently emphasized habitat amount and connectivity (Chase et al., 2020; Fahrig, 2013, 2020; Fletcher et al., 2023; Riva & Fahrig, 2022, 2023), the natural occurrence of wildfires in the region poses significant challenges to this goal because boreal landscapes are dynamic and continually changing. For example, a single large wildfire might alter patch configuration or habitat amount for a species over hundreds of square kilometers, while also shifting the relative proportion of available habitats (Erni et al., 2017). Although prioritizing habitat amount and connectivity may conserve biodiversity across many biomes currently undergoing habitat loss, this approach tends to downplay the relative importance of deterministic factors such as habitat type, habitat diversity, and species traits (but see Riva & Fahrig, 2023) that influence biodiversity at local and landscape scales (MacArthur & MacArthur, 1961; Thomsen et al., 2022). As such, conservation paradigms such as the habitat patch concept (sensu Fahrig, 2013) that are designed to mitigate the effects of habitat loss alone may be less appropriate in ecosystems where wildfire is a dominant feature.

The pyrodiversity–biodiversity hypothesis posits that spatial and temporal variation in the characteristics of wildfire promotes biodiversity by maintaining habitat heterogeneity (Jones et al., 2022; Jones & Tingley, 2021; Martin & Sapsis, 1992; Steel et al., 2024). Under this hypothesis, a landscape with high pyrodiversity has greater habitat and niche space diversity and therefore facilitates coexistence of a wider range of species (Burrows, 2008; Martin & Sapsis, 1992; Parr & Brockett, 1999). The notion that recent human activities (e.g., fire suppression, loss of indigenous fire management) may be decreasing pyrodiversity in some landscapes is a central theme of the hypothesis (Jones & Tingley, 2021; Martin & Sapsis, 1992), with proponents arguing in favor of restoring the spatiotemporal patterns of wildfire to conserve biodiversity in fire‐prone ecosystems (Ponisio et al., 2016; Tingley et al., 2016; but see Parr & Anderson, 2006; Taylor et al., 2012). Numerous studies have evaluated this hypothesis in forested ecosystems where the relationships between pyrodiversity and biodiversity were positive (Cohn et al., 2015; Ponisio et al., 2016; Sitters et al., 2014; Tingley et al., 2016), or neutral (Burgess & Maron, 2016; Prowse et al., 2017; Wills et al., 2020), yet few studies have tested the hypothesis in the boreal forest (but see Stuart‐Smith et al., 2002) despite fire regimes in this region undergoing rapid change (Cunningham et al., 2024; Hanes et al., 2019; Sayedi et al., 2024).

**FIGURE 1:**
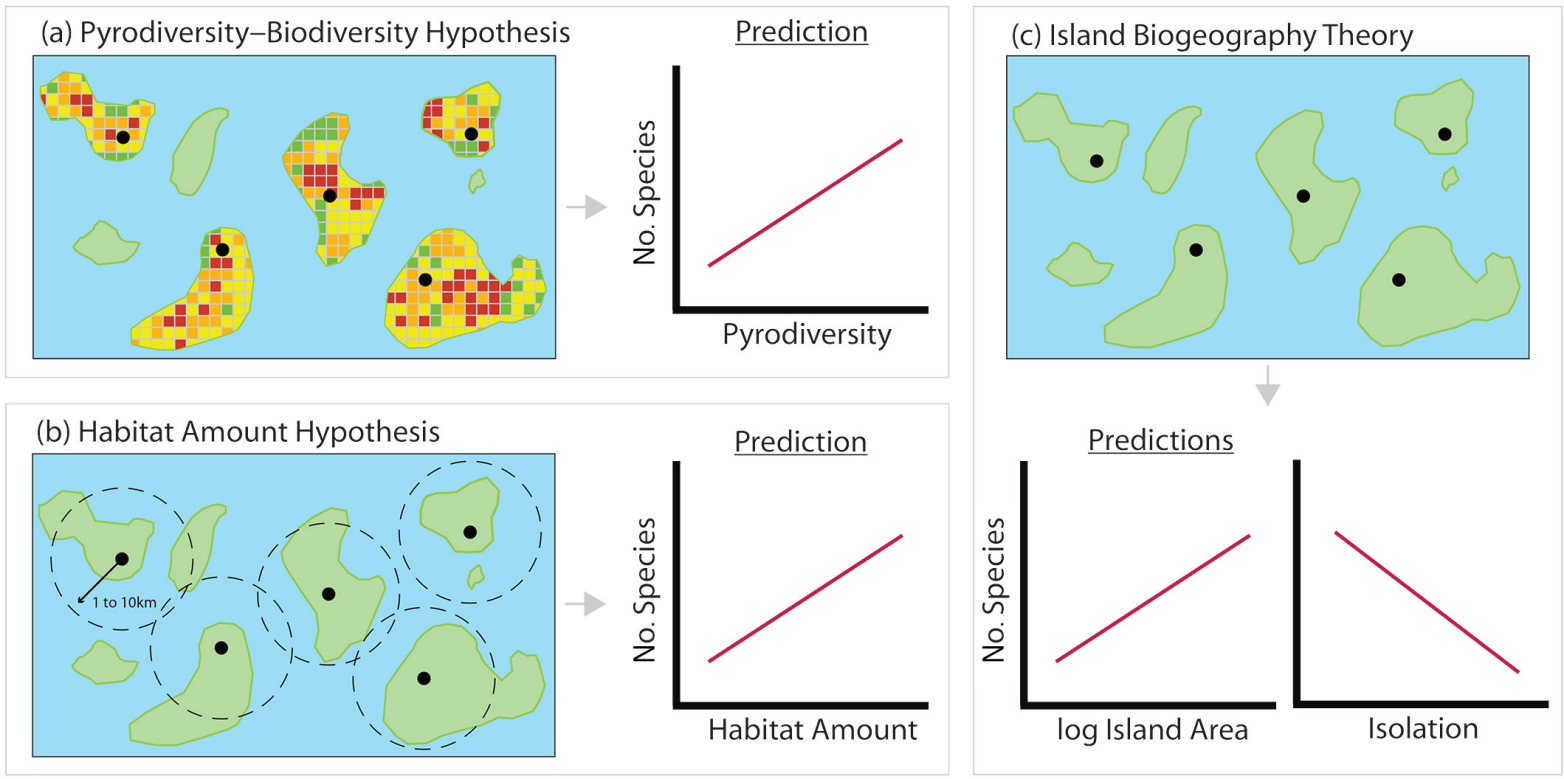
Illustration of the three hypotheses tested in our study and their associated predictions. (a) The pyrodiversity–biodiversity hypothesis proposes that variation in the spatial and temporal characteristics of wildfire increases habitat heterogeneity, and by extension, biodiversity. Variation in burn severity on each island (i.e., spatial pyrodiversity) was measured using the SD of relativized burn ratio (RBR) values (red squares: high burn severity, yellow and orange squares: intermediate burn severity, and green squares: low burn severity) with the expectation that species richness increases on islands with higher spatial pyrodiversity. (b) The habitat amount hypothesis predicts that species richness at a sampling site increases with the amount of habitat in the local landscape, where habitat (i.e., land) is measured within multiple spatial extents (1‐km increments up to 10 km) surrounding each sampling site, including the contribution of nearby islands or mainland within those extents. (c) The island biogeography theory posits that, based on data sampled from equal‐sized island plots (see Gotelli & Graves, 1996; Kelly et al., 1989), species richness increases with island area and decreases with increasing isolation.

Here, we tested the pyrodiversity–biodiversity hypothesis (Figure 1a) in a lake island archipelago and compared its ability to explain species richness patterns on true islands with two other theoretical frameworks commonly used to guide conservation planning on the mainland: the habitat amount hypothesis (Figure 1b, Fahrig, 2013) and island biogeography theory (Figure 1c, MacArthur & Wilson, 1963, 1967). The habitat amount hypothesis predicts that species richness should increase with the amount of habitat in the local landscape, as defined by an appropriate distance around the study site (Fahrig, 2013). In contrast, island biogeography theory posits that island species richness is the product of a dynamic equilibrium between local extinction and immigration rates and that species richness (both on the whole island, and per unit fixed area) increases with island area and decreases with isolation (MacArthur & Wilson, 1963, 1967). Through the use of 19 lake islands that burned between 1995 and 2021, we measured within‐island spatial pyrodiversity (i.e., SD of fire severity scores) from Landsat imagery and tested which of these three hypotheses provided a more parsimonious explanation for species richness (alpha diversity) patterns of beetles, plants, and birds (Figure 1). Using ecological gradients of island area, isolation, and time since fire constructed from a larger dataset of 42 islands, we also tested whether pairwise differences among islands in time since fire (i.e., temporal pyrodiversity) or in island area and isolation influence species turnover (beta diversity) across the archipelago.

## MATERIALS AND METHODS

### Study site

**FIGURE 2:**
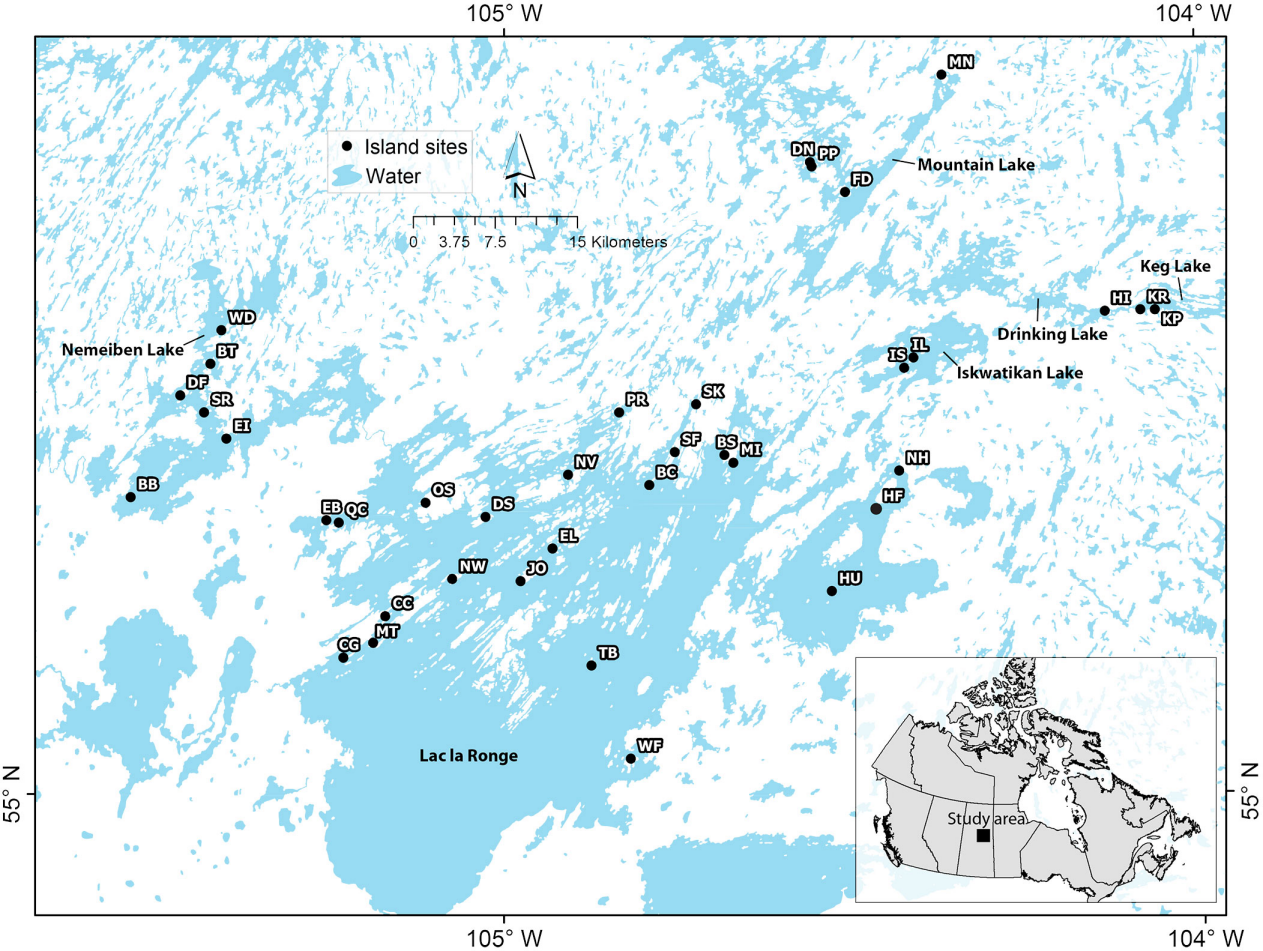
Map of 42 study islands in a freshwater boreal archipelago spanning three ecological gradients: area (1.0–380.7 ha), isolation (0.1–7.9 km from mainland), and time since fire (1–231+ years). Island sampling sites were located within six lakes within the Churchill River watershed including Lac la Ronge (*n* = 27 islands), Nemeiben Lake (*n* = 6), Mountain Lake (*n* = 4), Iskwatikan Lake (*n* = 2), Keg Lake (*n* = 2), and Drinking Lake (*n* = 1). Black circles indicate islands that were sampled. See Appendix S1: Table S1 for island information and abbreviations.

We conducted our study during 2019–2022 on 42 pristine lake islands in the Lac la Ronge region (55.22°, −104.99°) of the Churchill River watershed in northern Saskatchewan, Canada (Figure 2). Islands in the region are of glacial origin (10,200–9000 years BP, Teller & Leverington, 2004), shallow‐soiled, and covered with mixed and conifer‐dominated forests typical of the boreal shield ecozone (Ecosites BS9, BS10, and BS11, McLaughlan et al., 2010). More details of the study area are presented in Bell et al. (2017a, 2017b). Wildfires in the region are typically stand‐renewing and occur less frequently on islands than on the nearby mainland, where the fire return interval is 99–104 years (Parisien et al., 2004) owing to their insularity and the “firebreak” effect of large lakes in the region (Nielsen et al., 2016). Furthermore, wildfires generally occur more frequently on islands with increasing proximity to mainland regions as these islands are more susceptible to ignition from embers originating from nearby mainland fires. Islands in the region have never been commercially harvested or salvage logged after wildfire.

### Island selection and fire history reconstruction

We selected islands with the goal of establishing environmental gradients of time since fire (1–231+ years), island area (1.0–350.4 ha), and isolation (0.1–7.9 km from mainland); see Appendix S1: Table S1. Fire history for the islands was reconstructed using a combination of Landsat imagery (fires from 1984 to present) and dendrochronology (fires pre‐1981). Although provincial and federal forest fire databases rarely document small fires (i.e., <200 ha) that are in the size range of our study islands, we used these data (SPSA 2019) to help assign accurate burn dates based on fire spotting on the adjacent mainland for several islands (HI, KP, KR, and MN) that burned pre‐Landsat. Landsat imagery was obtained from GloVis (http://glovis.usgs.gov) and used to construct a composite image of the Lac la Ronge region using bands 3, 4, and 5 for Landsat 4 and 5, and bands 4, 5, and 6 for Landsat 8. Composite images were then used to identify prospective islands that had burned, and the existence of a fire was verified by comparing imagery of the same location 1 year prior. Although the large number of lake islands in our study system made it possible to reconstruct these three ecological gradients, time since fire and isolation were positively correlated (*r* = 0.65, *p* = <0.001) due to the natural refugia effect of surrounding water and paucity of recent burns among the most isolated islands (Nielsen et al., 2016).

We established a single 30 by 30 m plot in forested habitat on each island and positioned it so that the edge of the plot was at least 15 m from the water's edge. Based on a single sampling plot of equal size for each island, island biogeography theory predicts a positive correlation between species richness per unit area and island size, and an inverse relationship between species number and isolation (Figure 1c, Kelly et al., 1989; Kohn & Walsh, 1994; Gotelli & Graves, 1996; Wardle et al., 2003; Jonsson et al., 2009). The habitat amount hypothesis predicts that the number of species in a fixed‐size plot increases with the total habitat area in the “local landscape” surrounding the plot (Fahrig, 2013). Furthermore, it predicts that the size of the patch in which the plot is located has no effect on species richness beyond its contribution to the total amount of habitat in its landscape (Fahrig, 2013; Watling et al., 2020). Here, we consider island size as analogous to patch size, and our measure of habitat amount around each plot includes the island sampled and potential contributions of nearby islands or mainland within the scale of effect (see below).

For islands that burned pre‐1981, tree cores (two cores per tree, positioned at least 90° apart) were collected at breast height from 25 trees (≥4 cm diameter) closest to the center of the plot using an increment borer. Cores were placed in plastic straws for transport back to the lab where they were dried, mounted, and sanded with progressively finer grit sandpaper to help improve visibility of annual rings (Speer, 2010). Annual rings were counted under a dissecting microscope, and the date of the oldest tree at each site was used as an estimated date of most recent fire for each island (Appendix S1: Figure S1). Two islands had a single tree that was considerably older than the next oldest tree in the plot (NW and TB, see Appendix S1: Figure S1), and for these islands, we performed our analysis using both the oldest and second oldest tree in the plot. We used a stand‐age reconstruction approach to wildfire history because most wildfires in our region are stand‐renewing (Parisien et al., 2004), and extensive surveys of the islands did not locate any fire scars.

### Sampling protocol

Beetles were sampled continuously between 26 May and 4 September in 2020 and 31 May and 23 August 2022 using a combination of pitfall and window traps. We installed five of each trap type per island with traps placed at the center and four corners of each 30 m by 30 m island plot, with a minimum distance of 15 m between traps of the same type to ensure independence of captures (Digweed et al., 1995). Window traps were constructed using a cloth funnel and collecting cup suspended beneath a plexiglass panel (20 × 30 cm, Hammond, 1997; Lee et al., 2015). Window trap panels were oriented in a random direction (using random uniform numbers between 0 and 360) and secured to a cable suspended at breast height between nearby trees, with at least 1 m between any tree and trap. A pitfall trap was positioned within 1 m of being directly below each window trap and dug into the ground so that the trap edge was flush with the surrounding substrate. Pitfall traps consisted of a larger 1‐L cup with small holes cut into the bottom to improve drainage and a smaller 500‐mL inner cup for ease of sample extraction (Spence & Niemelä, 1994). A wooden lid (15 × 15 cm) was suspended above each pitfall trap to prevent rainwater and excess debris from clogging the trap. Approximately 100–150 mL of propylene glycol was added to the collecting vessel of each trap to help preserve samples until they could be collected. Traps on each island were visited at approximately 3‐week intervals (17–26 days, weather permitting) throughout the summer to collect samples and replenish trap preservative. See Appendix S1: Methods for additional details about beetle identifications and voucher locations.

Boreal songbird community composition was sampled during a single active breeding season (2 June to July 1) on each island plot between 2019 and 2022 using Wildlife Acoustics SM2+ Acoustic Recording Units (ARUs) with paired SMX‐II microphones (Wildlife Acoustics Inc.^©^, Maynard, MA). Microphones were tested using a sound‐level calibrator (Model 407744, Extech Instruments, Nashua, New Hampshire, USA) prior to deployment in the field (Turgeon et al., 2017) and only those sufficiently sensitive (i.e., ≥−44 dBV) were used. A single ARU was deployed at the center of each island plot by fixing it to a nearby tree approximately 1.5–2 m off the ground (Darras et al., 2018). ARUs were set to record at least one 3‐min recording per hour for the 6‐h duration of the dawn chorus (i.e., 1 h pre‐sunrise to 5 h post‐sunrise). Recordings were made in stereo .wav file format using a sampling rate of 44,100 samples per second with the microphone preamplifier gain settings set to factory default. A stratified random sample of six 3‐min recordings per island was then processed to provide a representative measure of the songbird community. Specifically, we created six temporal sampling strata based on the combination of time of day (early dawn chorus: 60 min prior to sunrise until 50 min after sunrise; mid‐dawn chorus: 51–150 min after sunrise; and late dawn chorus: 151–300 min after sunrise) and time of the deployment (early vs. late relative to the median Julian date). ARU recordings were manually processed by expert listeners in the WildTrax online platform for managing, storage and processing of wildlife sensor data (https://www.wildtrax.ca/). Listeners were required to listen to recordings while using stereo headphones that fully covered the ears, had a minimum frequency response of 20–20,000 Hz, had a flat frequency response, could not be noise canceling, and used a combination of spectrogram and listening to draw “tags” around species‐individual identifications. Tags within WildTrax facilitate post hoc verification by ≥1 listener, and we selected the two listeners with the highest test scores in a qualifying examination to verify tags of the original listeners (McManus et al., 2024) using the species verification process within WildTrax. We removed any corvids, waterfowl, and raptors that we detected prior to analysis because these species were likely utilizing habitats (i.e., shoreline) outside of our forested island plots. We also acknowledge that not all bird species are detectable with passive acoustic monitoring.

Vascular plant species composition was assessed in July and August 2021 by recording and identifying all plant and tree species (Harms & Leighton, 2011; Johnson et al., 1995; Leighton, 2012; Leighton & Harms, 2020) within a 15‐m radius of the center of each island plot. Plant species composition was assessed for all islands except island BS, where our study site burned in early 2021 before the survey could be conducted. Representative specimens of sedges and grasses were collected, pressed, and transported to the lab where they were identified under 20× magnification. In addition to these surveys of plant species presence/absence, we recorded information on forest canopy and understory structure using the line‐intercept method (0.1‐m increment) for all woody species (understory, <4 cm dbh (~1.3 m), < 2 m in height; overstory, ≥4 cm diameter, >2 m in height) along three 30‐m transects within each island plot, positioned 15 m apart. The length and diameter of all fallen or leaning (≥4 cm diameter) coarse woody debris (CWD) on these transects and their decay class (DC 1–5, e.g., fresh deadwood = DC 1) was recorded. Presence or absence of charcoal on all CWD was also recorded to distinguish between CWD that originated from wildfire (i.e., charcoal present) or from windthrow or tree senescence. Diameter was recorded at the central point of each piece of CWD, and volume (*V*) was estimated using the formula: *V* = π*r* ^2^ *h*, where *r* is the radius and *h* is the length of each piece of CWD. Lastly, we calculated basal area (i.e., standing woody biomass) for each island plot by measuring the dbh of all trees ≥4 cm dbh within a 10‐m radius of the center of each island plot.

### Island isolation and habitat amount

We developed an isolation index based on the proportion of water surrounding each island to capture the general clustering of islands in our study system and the potential for colonization between neighboring islands (Bell et al., 2017a). Compared to nearest distance measures, this approach accounts for the clustering effect of islands in our system and is more likely to detect significant effects of connectivity if they are present (Moilanen & Nieminen, 2002). To do this, we used GIS software to convert a vector shapefile (minimum mapping unit = 1.56 ha) of our study area to a binary water/land raster grid (1—water; 0—land) with a cell size of 5 m, applied a 5‐km buffer around the perimeter of each island, and used zonal statistics to calculate the proportion of water (vs. land) inside the buffer.

To quantify habitat amount, we classified habitat as land and non‐habitat as water and used a similar GIS approach to that used for our isolation index above, except in this case the binary raster grid was reversed (1—land; 0—water) and the buffer was centered on the island plot. This measure of habitat amount therefore incorporates the island where the plot was located and land in the local landscape (i.e., nearby islands or mainland). Furthermore, because the appropriate local landscape scale was unknown for the taxa in our system, we performed a multi‐scale analysis (see Fahrig, 2013) by comparing slopes of the relationship between species richness and habitat amount in buffers at 1‐km increments (1–10 km, see Appendix S1: Figure S2). We selected the scale (i.e., buffer distance) for each of the three taxa which produced the strongest slope in the relationship between richness and habitat amount and used this scale in the analysis evaluating the habitat amount hypothesis (Table 1, Appendix S1: Figure S2).

**TABLE 1:** List of candidate regression models reflecting the underlying predictions of the island biogeography theory, habitat amount hypothesis, and pyrodiversity–biodiversity hypothesis, their support in explaining species richness patterns of beetles, plants, and birds on 19 islands, model structure, number of parameters (*K*), deviance explained (*D* ^2^), Akaike information criterion with small sample correction (AIC_c_) scores, differences in AIC_c_ scores between each model and the most supported model (Δ_*i*_), and AIC_c_ weights (*w*_*i*_) of each model.

Species type and model no.	Model name	Model structure	*K*	*p*	*D* ^2^	AIC_c_	Δ_*i*_	*w*_*i*_
Beetles								
6	Habitat amount + Pyro model	Model 3 + Model 4	5	<0.001	0.26	167.09	0	0.90
4	Pyrodiversity model	sd.fire.severity + time.since.fire + time.since.fire^2^	4	<0.001	0.17	172.72	5.63	0.05
3	Habitat amount model	%land.buffer1.km	2	0.002	0.10	173.56	6.46	0.04
5	Island Biogeog. + Pyro model	Model 2 + Model 4	6	<0.001	0.21	176.72	9.62	0.01
2	Island Biogeog, model	log_10_(area + 1) + isolation index	3	0.030	0.07	179.14	12.04	0
1	Null model	1	1		0	180.69	13.59	0
Plants								
4	Pyrodiversity model	sd.fire.severity × time.since.fire + time.since.fire^2^	5	<0.001	0.34	124.92	0	0.67
5	Island Biogeog. + Pyro model	Model 2 + Model 4	7	<0.001	0.42	127.32	2.39	0.20
6	Habitat amount + Pyro model	Model 3 + Model 4	6	<0.001	0.36	128.26	3.33	0.13
2	Island Biogeography model	log_10_(area + 1) + isolation index	3	0.002	0.13	139.86	14.94	0
1	Null model	1	1		0	147.01	22.08	0
3	Habitat amount model	%land.buffer10.km	2	0.784	0.01	149.45	24.52	0
Birds								
4	Pyrodiversity model	sd.fire.severity + time.since.fire	3	0.003	0.11	103.23	0	0.39
5	Island Biogeog. + Pyro model	Model 2 + Model 4	5	0.001	0.18	103.94	0.71	0.27
2	Island Biogeography model	log_10_(area + 1) + isolation index	3	0.006	0.10	104.36	1.13	0.22
6	Habitat amount + Pyro model	Model 3 + Model 4	4	0.008	0.12	106.21	2.98	0.09
1	Null model	1	1		0	109.33	6.10	0.02
3	Habitat amount model	%land.buffer1.km	2	0.120	0.02	109.42	6.20	0.02

### Temporal and spatial pyrodiversity

**FIGURE 3:**
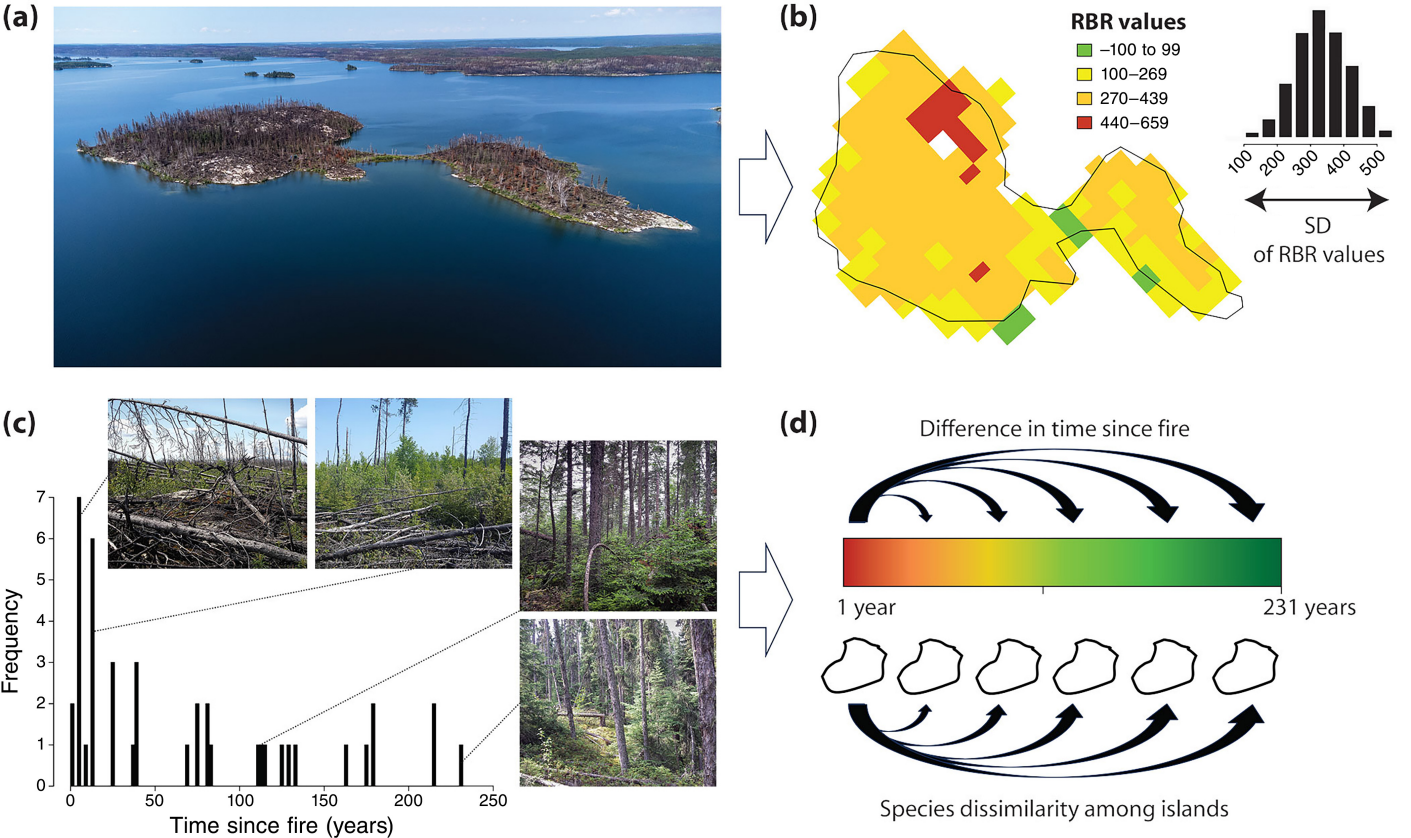
Representation of the two methods used to measure and quantify pyrodiversity for testing the pyrodiversity–biodiversity hypothesis. (a) Spatial pyrodiversity was calculated for the 19 islands that burned between 1995 and 2021 based on the variation in fire severity obtained from pre‐ and postfire analysis of Landsat imagery. (b) Fire severity is expressed as relativized burn ratio (RBR) values (30 m^2^ resolution, island HF depicted) with cells in green (RBR values <100) indicating unburnt areas with little vegetation change, whereas cells in yellow (100–439 RBR values) and red (RBR values >440) signify areas with low to higher fire severity, respectively, where the change in vegetation pre‐ and postfire was more substantial. Variation in fire severity across each island was measured using the SD of RBR values. (c) Using the islands that span our time since fire gradient, we estimated temporal pyrodiversity using (d) pairwise Euclidean distance (i.e., absolute difference in time since fire between island pairs) and tested if it was related to pairwise dissimilarity (beta diversity) of beetle, plant, and bird assemblages. Photo credit: (a) b/w Photo; (c) Aaron Bell.

We estimated the spatial and temporal components of pyrodiversity in our island system using two different approaches. First, we created burn‐severity rasters for the subset of 19 islands that burned between 1995 and 2021 in Google Earth Engine using code provided by Parks et al. (2019) (see Figure 3). This script uses changes in reflectance from pre‐ and postburn satellite imagery (30 m resolution) to estimate the magnitude of change in soil and vegetation following fire, expressed as relativized burn ratio (hereafter RBR, Parks et al., 2014, 2019). We then extracted RBR values from each fire severity raster for the full extent of each island using the *terra* package (Hijmans, 2023). Because large islands could have higher variation in RBR values owing to the larger area sampled (i.e., more RBR values on larger islands), we randomly sampled the original RBR values 10,000 times for each island (drawing one cell per sample, with replacement) and, from these data, used the SD of RBR values as our estimate of spatial pyrodiversity for each island (Tingley et al., 2016). Spatial pyrodiversity, in this case, is a measure of spatial variation in fire characteristics within each island (i.e., our sampling unit) that facilitates a direct test of the pyrodiversity–biodiversity hypothesis (see Jones & Tingley, 2021).

Using the gradient of time since fire, we also developed an estimate of temporal pyrodiversity using the full set of 42 islands. Here, we used pairwise differences in time since fire (i.e., Euclidean distance) among islands as our measure of temporal pyrodiversity (Anderson et al., 2011) and tested whether turnover in community structure (i.e., Jaccard's index for beetles, plants, and birds) was influenced by temporal pyrodiversity, or by similar pairwise spatial distances constructed from gradients of island area and isolation. In contrast to our measure of spatial pyrodiversity, variation in temporal pyrodiversity is considered among (and not within) our sampling units, with the expectation that greater variation in time since fire supports higher beta diversity (Jones & Tingley, 2021; Taillie et al., 2018). Although this latter approach is considered an indirect test of the pyrodiversity hypothesis (Jones & Tingley, 2021), the combined use of spatial (within islands) and temporal pyrodiversity (among islands) in our study emphasizes the importance and interdependence of processes occurring at the patch and landscape scale (see Fletcher et al., 2023).

### Data analysis

To ensure that our sampling effort adequately characterized the species assemblage on each island and that our species richness data were not biased by rare species that were present but undetected, we used multispecies occupancy models (MSOMs) to estimate species richness of beetles and birds after accounting for species‐level variation in detection (Dorazio et al., 2006; Dorazio & Royle, 2005). MSOMs link models of detection and occurrence for each species and facilitate community‐level assessments of species richness that are unbiased and more precise (Zipkin et al., 2010). The number of observation events at each site in our study was four for beetles and six for birds. We assumed that occurrence probability varied by island and that detection probability of beetles and birds was influenced by species traits. For beetles, we expected feeding guild and body size to influence microhabitat preferences, food resources, and movement that could affect detectability in pitfall and window traps (Sheehan et al., 2019; Work et al., 2002). For each species, body size information was compiled from the literature (see Appendix S1: Methods) or measured with calipers (tip of mandibles to apex of elytra) and each species was assigned to one of seven feeding guilds (carnivores, fungivores, herbivores, omnivores, palynivores, saprovores, and saproxylics) based on family‐level information in Rappa et al. (2022). For birds, we used information on mass, maximum vocal frequency, and residency (migrant vs. resident) obtained from the *lhreg* package in R (Sólymos, 2017) and Brigham and Fenton (1991) because these are characteristics known to influence auditory detection of birds (Sólymos et al., 2018). We developed MSOMs in the R package *RPresence* (Donovan et al., 2023; MacKenzie & Hines, 2025) and evaluated models reflecting combinations of these detection covariates using Akaike information criterion (AIC, Appendix S1: Table S2). The most supported model (lowest AIC) was then used to estimate beetle and bird species richness for use in our model selection (see below).

Island species richness was analyzed for the subset of 19 islands using generalized linear regression models with a Poisson error distribution and log link function to avoid biased estimation associated with commonly used transformations (O'Hara & Kotze, 2010). To test whether the pyrodiversity–biodiversity hypothesis, island biogeography theory, or habitat amount hypothesis best explained species richness patterns on the islands, we used a multi‐hypothesis framework comparing a priori models reflecting the underlying predictions of the three theories (Table 1, MacArthur & Wilson, 1963, 1967; Martin & Sapsis, 1992; Fahrig, 2013). Candidate models were evaluated using AIC with small sample correction (AIC_c_) and the most supported model had the smallest AIC_c_ and largest Akaike weight (*w*_*i*_) (Anderson et al., 2000). For models assessing support for the pyrodiversity–biodiversity hypothesis, we included time since fire and used a model selection procedure to determine if the three taxa responded to this variable as a linear or quadratic predictor. Given the well‐documented interaction between the effects of time since fire and fire severity (Taillie et al., 2018; Tingley et al., 2016), we also tested for potential interactions between time since fire and spatial pyrodiversity variables in our pyrodiversity models. We similarly tested for an interaction between island area and isolation index but found no support for such an interaction, so we removed it from our biogeography models. Due to minor variation in beetle sampling effort that resulted from animals disturbing pitfall and window traps, we included the logarithm of total number of trapping days for each island as an offset in our models for beetle richness. Lastly, we checked the residuals of the most supported models using spline correlograms created with the *ncf* package (Bjornstad, 2022) to ensure that our analyses were not affected by spatial autocorrelation.

We assessed statistical significance of variables in our models using incidence rate ratio (IRR) and 95% CIs, with CIs that did not overlap a value of one indicating variables that were significant. We also calculated *p*‐values (*p*) for models using a likelihood ratio χ^2^ test comparing candidate models to a null model with a fixed intercept. Although some researchers have argued against inference combining null hypothesis testing (*p*‐values) and information‐theoretic approaches (AIC, effect sizes) based on theoretical grounds (Anderson & Burnham, 2002; Lukacs et al., 2007), others have defended a more pluralistic stance (Castilho & Prado, 2021; Stephens et al., 2005, 2007). We used both approaches in our statistical inference to help facilitate comparisons between results that were analyzed using all 42 islands and results from our AIC‐based model selection that was limited to a subset of 19 islands for which fire severity data were available.

We analyzed beta diversity for the full set of 42 islands to test whether differences in island attributes (area or isolation) or temporal pyrodiversity influenced community composition among the islands. To do this, we used multiple regression models for distance matrices to explore relationships between Jaccard's similarity index (beta diversity) and the Euclidean distance matrices of island area, isolation, and time since fire (Legendre & Legendre, 2012; Si et al., 2015). Due to the lack of independence between island pairs (as each island appears in multiple pairs), we used Mantel tests (999 permutations) to estimate the *p*‐values and Pearson correlation coefficients for each model. These analyses were conducted using the *vegan* and *ecodist* packages (Goslee & Urban, 2007; Oksanen et al., 2020) in R version 4.1.1 (R Core Development Team, 2021).

To explore potential mechanistic explanations for how temporal pyrodiversity influences beta diversity, we used redundancy analysis (RDA) and a step function model selection procedure to explore what local habitat variable(s) (Appendix S1: Table S3) best explained heterogeneity in beetle, plant, and bird assemblages based on AIC. Island BS was omitted from the RDA analysis because it burned before plant surveys could be conducted. Local habitat variables (Appendix S1: Table S3) were standardized prior to the selection procedure and variance inflation factor (VIF) was used afterward to assess model covariates for multicollinearity. No variables included in our final models showed an influence of multicollinearity (VIF < 10, Chatterjee et al., 2000). All analyses were conducted in R statistical software (R Core Development Team, 2021).

## RESULTS

### Alpha diversity

We detected a total of 621 unique species on the 42 islands sampled, representing 466, 101, and 54 species of beetles, plants, and birds, respectively (Appendix S1: Tables S4 and S5; see also Beetle_species_list.xlsx in Bell & Van Wilgenburg, 2026). Model selection for our multispecies occupancy analysis showed that for both beetles and birds, the most supported model included all the species‐level detection covariates we evaluated (Appendix S1: Table S2). Furthermore, our estimated species richness (MSOM‐adjusted) from this analysis was highly correlated with sampled species richness for beetles (*n* = 42, *R* ^2^ = 0.99, *p* = <0.001, species richness_MSOM_ = 2.32 [95% CI = 0.83–3.82] + β = 1.26 [CI = 1.25–1.28]) and birds (*n* = 42, *R* ^2^ = 0.99, *p* = <0.001, species richness_MSOM_ = 0.19 [CI = −0.19 to 0.56] + β = 1.13 [CI = 1.10–1.17]), suggesting that our sampling procedure adequately characterized species assemblages on the islands and that our measures of species richness were not biased by poorly detected species. Finally, residual variation between sampled and estimated species richness was unrelated to island area, isolation, and habitat amount (Appendix S1: Figure S3). Hereafter, all results for species richness of beetles and birds refers to MSOM‐adjusted species richness.

**FIGURE 4:**
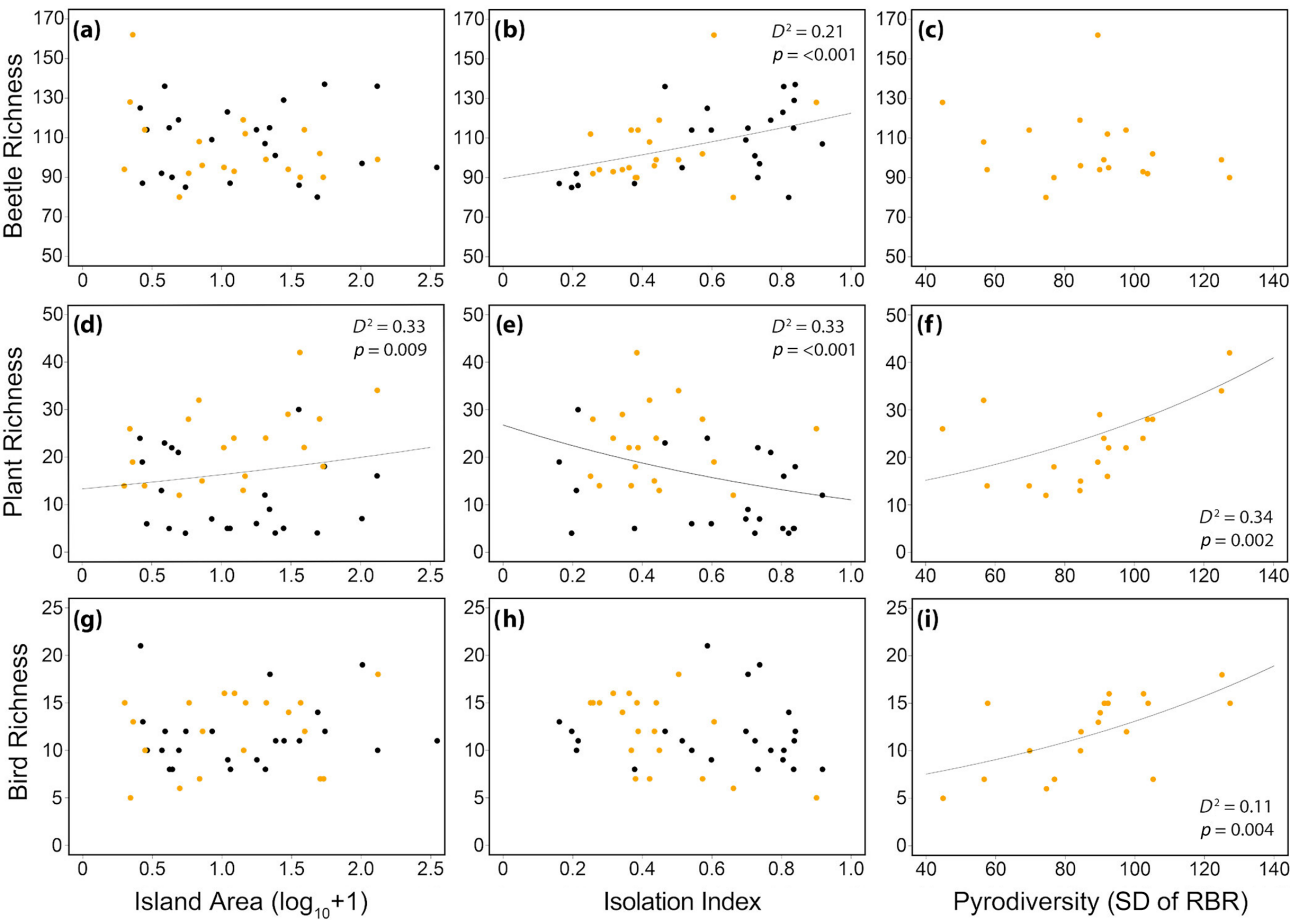
Regression plots of species richness (alpha diversity) against island area, isolation, and spatial pyrodiversity for beetles (a–c), plants (d–f), and birds (g–i) on the islands in our study. Data points in orange indicate the subset of 19 islands that were included in our model selection evaluating support for the island biogeography theory and pyrodiversity–biodiversity hypothesis (island burned between 1995 and 2021), whereas data points in black indicate islands that burned pre‐1995. Island isolation index was measured as the proportion of water around each island within 5‐km buffers, with islands approaching a value of 1 being more isolated, and values nearing 0 less isolated. The predicted line in the plots includes other model covariates set at the mean (see Table 1). Deviance explained (*D* ^2^), *p*‐value (*p*) of significant explanatory variables, and SD of relativized burn ratio (RBR).

For models using all 42 islands, species richness was not related to island area for beetles (*n* = 42, *p* = 0.517, IRR = 0.99 [CI = 0.96–1.02], Figure 4a, Appendix S1: Table S6) or birds (*n* = 42, *p* = 0.186, IRR = 1.06 [CI = 0.97–1.16], Figure 4g), but significantly increased with island area for plants (*n* = 42, *p* = 0.009, IRR = 1.12 [CI = 1.03–1.22], Figure 4d). Plant richness also significantly decreased with isolation (*n* = 42, *p* = <0.001, IRR = 0.80 [CI = 0.74–0.87], Figure 4e) as predicted by island biogeography theory, whereas beetle richness increased with isolation (*n* = 42, *p* = <0.001, IRR = 1.07 [CI = 1.04–1.10], Figure 4b) and bird richness was unrelated to isolation (*n* = 42, *p* = 0.058, IRR = 0.92 [CI = 0.83–1.00], Figure 4h). The variable and contrasting effects of island spatial variables was also reflected in our model selection analysis using a subset of 19 islands (Table 1), where support for the island biogeography model was equivalent to the null model for both beetles (*n* = 19, *D* ^2^ = 0.07, *p* = 0.033, *w*_*i*_ = <0.01) and plants (*n* = 19, *D* ^2^ = 0.13, *p* = 0.002, *w*_*i*_ = <0.01). Although the island biogeography model alone explained some variation in bird species richness (*n* = 19, *D* ^2^ = 0.10, *p* = 0.006, *w*_*i*_ = 0.22), two of the five other models we evaluated had greater support (Table 1).

Habitat amount was highly correlated with our isolation index at both 10‐km (Pearson's *r* = −0.92, *p* = <0.001) and 1‐km scales (*r* = −0.62, *p* = <0.001, Appendix S1: Figure S4), and consequently our analysis of species richness in relation to these variables yielded similar results. When considering all 42 islands, habitat amount was positively related to species richness for plants (*n* = 41, *p* = <0.001, IRR = 1.28 [CI = 1.18–1.38], Appendix S1: Figure S5), unrelated for birds (*n* = 42, *p* = 0.061, IRR = 1.09 [CI = 1.00–1.19]), and negatively related for beetles (*n* = 42, *p* = <0.001, IRR = 0.93 [CI = 0.90–0.96]). Model selection based on the subset of 19 islands suggested that there was generally low support for the habitat amount hypothesis alone, which was equivalent or worse than the null model (Table 1) for both plants (*n* = 19, *D* ^2^ = 0.01, *p* = 0.784, *w*_*i*_ = <0.01) and birds (*n* = 19, *D* ^2^ = 0.02, *p* = 0.120, *w*_*i*_ = 0.02). Although there was greater support for the habitat amount hypothesis for beetles (*n* = 19, *D* ^2^ = 0.10, *p* = 0.002, *w*_*i*_ = 0.04), and the most supported model for beetles included habitat amount and pyrodiversity models combined (*n* = 19, *D* ^2^ = 0.26, *p* = 0.002, *w*_*i*_ = 0.90, Table 1), the observed negative relationship between beetle richness and habitat amount (Appendix S1: Table S6) contrasts with predictions of the hypothesis.

Of the three a priori models we assessed to explain species richness patterns on the islands, the pyrodiversity model had the greatest support for plants (*n* = 19, *D* ^2^ = 0.35, *p* = <0.001, *w*_*i*_ = 0.67) and birds (*n* = 19, *D* ^2^ = 0.11, *p* = 0.003, *w*_*i*_ = 0.39, Table 1). The pyrodiversity model was also the second‐most supported model for beetles (*n* = 19, *D* ^2^ = 0.17, *p* = <0.001, *w*_*i*_ = 0.05, Table 1), although the effect of spatial pyrodiversity on beetle richness was not significant (*n* = 19, *p* = 0.433, IRR = 1.02 [CI = 0.97–1.07], Figure 4c) and support for this model was driven by effects of time since fire on beetle richness (*n* = 19, *p* = <0.001, IRR = 0.90 [CI = 0.85–0.94], Appendix S1: Table S6). In contrast, we observed significant positive effects of spatial pyrodiversity on both plant (*n* = 19, *p* = <0.001, IRR = 1.24 [CI = 1.11–1.39], Figure 4f) and bird (*n* = 19, *p* = 0.004, IRR = 1.22 [CI = 1.07–1.39], Figure 4i) species richness including support for an interaction between time since fire and spatial pyrodiversity for plants (*n* = 19, *p* = 0.020, IRR = 0.85 [CI = 0.74–0.98], Appendix S1: Table S6).

### Beta diversity

**FIGURE 5:**
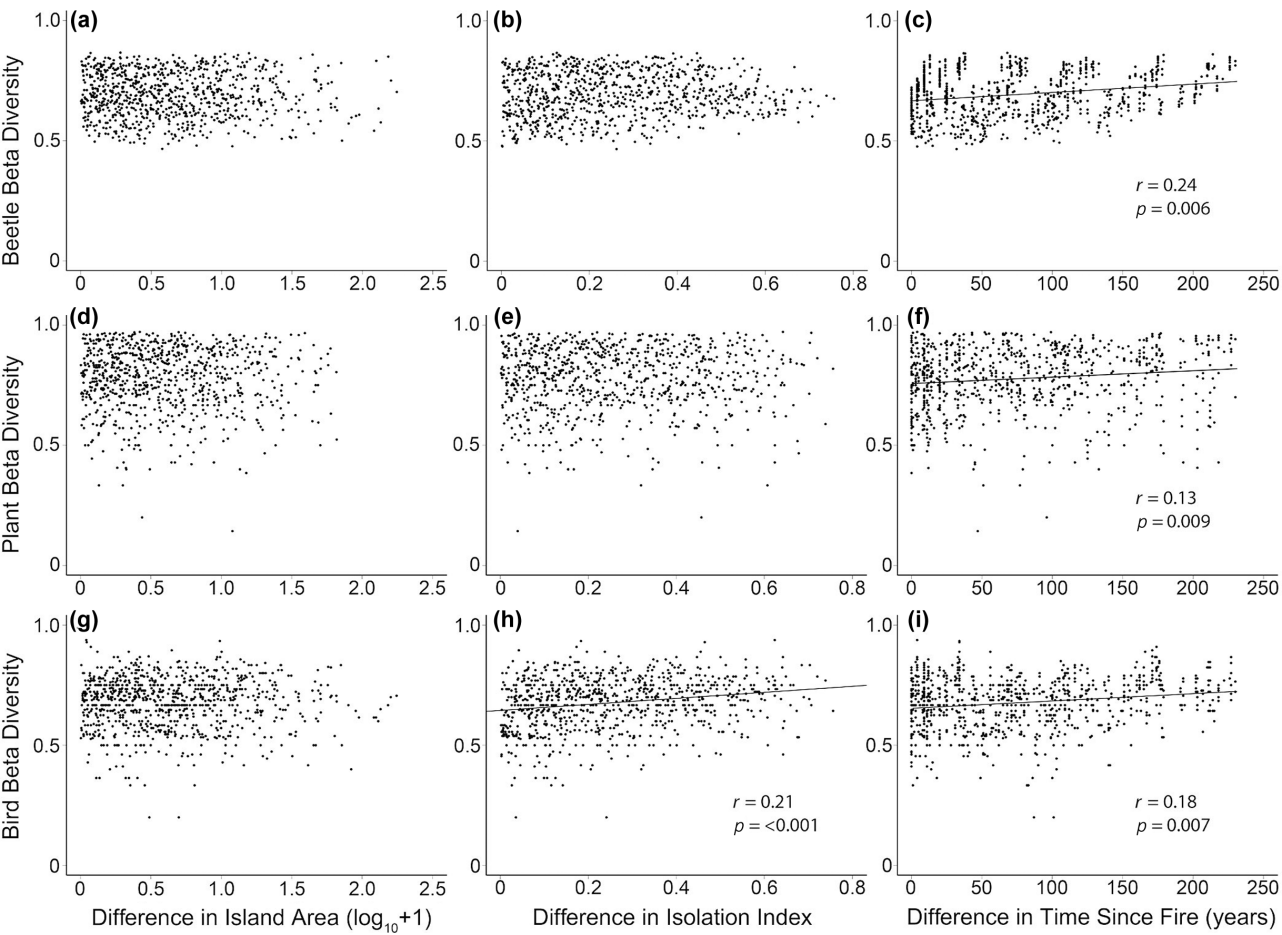
The relationship between beta diversity (Jaccard's index) and absolute differences in island area, isolation index, and time since fire (i.e., temporal pyrodiversity) for beetles (a–c), plants (d–f), and birds (g–i) among islands. The mantel statistics (*r*) and *p*‐value (*p*) for testing correlations between the dissimilarity matrices.

Beta diversity did not vary with pairwise differences in island area for any of the three taxa (beetles *r* = −0.02, *p* = 0.580; plants *r* = −0.02, *p* = 0.659; birds *r* = −0.01, *p* = 0.543; Figure 5a,d,g), suggesting that in general large and small islands were similar in overall species composition. Similarly, beta diversity did not vary with pairwise differences in isolation for either beetles (*r* = 0.05, *p* = 0.192, Figure 5b) or plants (*r* = 0.02, *p* = 0.252, Figure 5e), suggesting that the effects of isolation and extent to which islands are clustered did not correspond to differences in species composition for these groups. In contrast, bird beta diversity was positively related to differences in isolation (*r* = 0.21, *p* = <0.001, Figure 5h).

**FIGURE 6:**
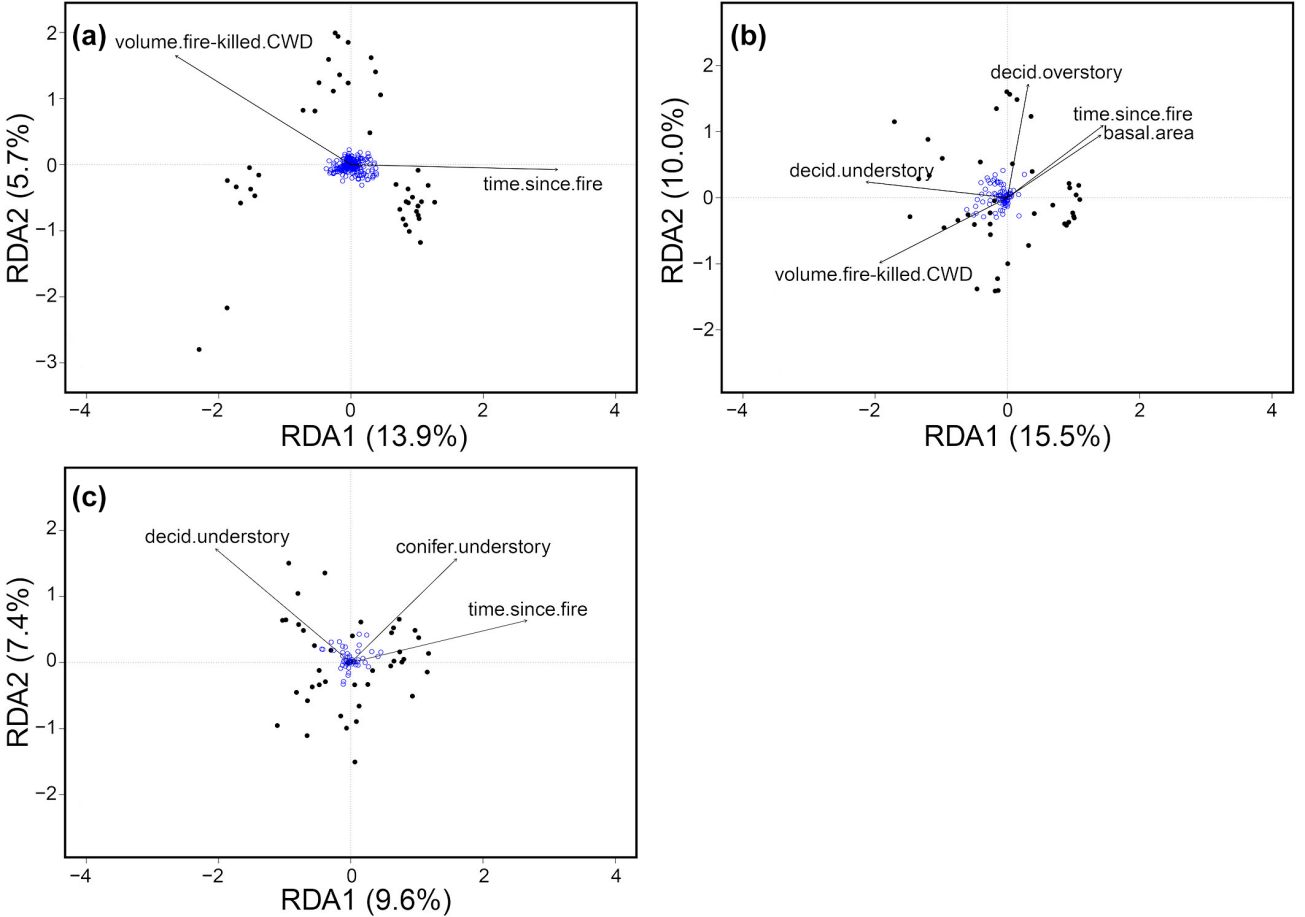
Constrained redundancy analysis (RDA) showing the ordination of beetle (a), plant (b), and bird (c) assemblages with significant habitat variables on 41 lake islands in north central Canada. Black points represent island sites and blue circles denote centroids of species. The arrow length of vectors indicates the relative weight of a given variable in the ordination and the direction denotes the correlation of that variable with each axis. See Appendix S1: Table S3 for the full list of candidate variables. CWD, coarse woody debris.

Beta diversity was positively related to temporal pyrodiversity for all three taxa (beetles *r* = 0.24, *p* = 0.006; plants *r* = 0.13, *p* = 0.009; birds *r* = 0.18, *p* = 0.007, Figure 5c,f,i), suggesting that landscape‐scale variation in fire history is an important factor influencing beetle, plant, and bird community composition. Exploration of the effects underlying variation in species composition in our constrained RDA analysis (Figure 6a) extracted two significant factors (axes) for beetles, that is, time since fire (*F* = 6.57, *p* = 0.001) and the volume of fire‐killed CWD (*F* = 2.68, *p* = 0.001). Meanwhile, our constrained RDA analysis for plants (Figure 6b) indicated that there were five significant factors influencing species composition including time since fire (*F* = 5.86, *p* = 0.001), volume of fire‐killed CWD (*F* = 5.18, *p* = 0.001), basal area (*F* = 1.86, *p* = 0.031), and percent cover of deciduous understory (*F* = 4.02, *p* = 0.001) and overstory (*F* = 3.18, *p* = 0.001). Finally, our constrained RDA for birds (Figure 6c) indicated that time since fire (*F* = 4.22, *p* = 0.001) and percent cover of deciduous (*F* = 3.05, *p* = 0.001) and conifer (*F* = 1.96, *p* = 0.007) understory shrub vegetation were significant factors influencing bird species composition. Overall, these findings demonstrate that species composition of all three taxa was strongly influenced by both temporal and spatial characteristics of wildfire.

## DISCUSSION

Wildfire regimes have been altered by human activities on a global scale and are predicted to intensify in the coming century (Cunningham et al., 2024; Sayedi et al., 2024), raising concerns about potentially large changes in wildfire patterns that maintain biodiversity in fire‐prone ecosystems (Erni et al., 2017; He et al., 2019). Our findings support the pyrodiversity–biodiversity hypothesis and the notion that biodiversity in the boreal forest is strongly influenced by spatiotemporal patterns of wildfire. In contrast, we found little support for the traditional view that island spatial properties such as area, isolation, or habitat amount are primary determinants of species richness for either beetles or birds. Although plant species richness on our islands initially appeared consistent with predictions from island biogeography theory and the habitat amount hypothesis, a post hoc analysis controlling for the negative effects of time since fire (Appendix S1: Results) revealed that area, isolation, and habitat amount had no significant effects. This helps to explain why our pyrodiversity model, which included the effects of time since fire, outperformed more complex models that incorporated pyrodiversity, island spatial properties, and habitat amount (e.g., Models #5 and 6, Table 1). The lower plant richness observed in plots on small, isolated islands with little surrounding habitat was instead driven by older island communities (i.e., >100 years since fire), which also tend be farther from the mainland (i.e., isolated) and less frequently exposed to fire (see Materials and Methods, Nielsen et al., 2016). We also found that pyrodiversity alone better explained the observed patterns in species richness for beetles and birds (Table 1), suggesting that wildfire‐mediated habitat heterogeneity is more important for biodiversity than are the physical dimensions and connectedness of patches within the boreal forest mosaic. In view of changing fire regimes and projected increases in wildfire intensity (Hanes et al., 2019; Wang et al., 2017), our findings indicate that efforts to sustain biodiversity in the boreal forest should consider how best to maintain pyrodiverse landscapes.

In their recent review, Jones and Tingley (2021) showed that support for the pyrodiversity–biodiversity hypothesis varied by taxa, biome, scale examined, and how pyrodiversity was measured or defined. In our study, we found notable congruence between two metrics (alpha diversity and Jaccard's index) of biodiversity across three separate taxa and found a generally positive relationship to pyrodiversity. However, our results also showed that taxa responded to different facets of habitat heterogeneity that underpin our measures of pyrodiversity. For example, beta diversity of plants was linked to deciduous overstory cover, but beta diversity of birds was related to both deciduous and coniferous shrub coverage in the understory (Figure 6b,c). Canopy cover may have influenced microclimate and light availability for understory plants (Swanson et al., 2011), whereas for birds understory shrub cover and differences in forest type likely affected availability of both nesting and foraging sites (Bayne et al., 2010; Hobson & Bayne, 2000). Similarly, while both plant and bird richness increased with spatial pyrodiversity, beetle richness did not, meaning that variation in burn severity and the resulting heterogeneity on our islands benefited some but not all groups. Previous studies have demonstrated that volume and quality (i.e., decay stage) of deadwood is linked to beetle species richness (Lassauce et al., 2011; Lee et al., 2014), suggesting that the extent of tree mortality at our sites (i.e., volume of fire‐killed woody debris) was more important for beetles than was the variation in the structure of living vegetation after fire. The strong influence of time since fire and volume of fire‐killed deadwood on beetle beta diversity in our study (see Figure 6a) further suggests that beetles are more responsive to the temporal dynamics of pyrodiversity, as opposed to its spatial patterns. Collectively, our findings, and those of others (He et al., 2019; Kelly & Brotons, 2017), support the view that diversity in the responses of species to habitat heterogeneity is the foundational mechanism for how pyrodiversity begets biodiversity.

Despite the consensus among researchers that wildfire plays a crucial role in maintaining ecosystem structure and function (Pausus & Keeley, 2009; Driscoll et al., 2010; but see Taylor et al., 2012), wildfires are often portrayed in the context of their destructive effects and the threat posed to private property (Paveglio et al., 2011). Our study underscores the crucial role that wildfires play in maintaining biodiversity and highlights the occurrence of unique fire‐adapted invertebrates that are rare or absent in unburnt habitats. For example, the two islands we sampled in the year immediately following fire recorded 37 beetle species not detected on any of the other 40 islands, which ranged from 5 to 231 years postfire (see Beetle_species_list.xlsx in Bell & Van Wilgenburg, 2026). Furthermore, at least four of these beetle species (Cryptophagidae: *Henoticus serratus*, Buprestidae: *Melanophila acuminata*, Carabidae: *Sericoda bembidioides*, and *Sericoda obsoleta*) are “pyrophilous” and rarely occur, if ever, outside of burnt habitats (Wikars, 1997; see review by Bell, 2023). Pyrophilic insects, a group made up of mostly beetles and flies, are likely dependent on recurrent wildfires and possess a range of adaptations reflecting long‐term co‐evolution with wildfire (Bell, 2023; Bell et al., 2022; Jones et al., 2023; Schmitz et al., 2016). However, previous studies evaluating the pyrodiversity–biodiversity hypothesis among invertebrates have primarily focused on non‐pyrophilous groups such as ants, termites, spiders, wasps, bees, and flies (see references in Jones & Tingley, 2021). Although pyrophilic insects clearly depend on temporal characteristics of wildfires, future studies examining their response to spatial heterogeneity in burn severity are warranted (Bell et al., 2022).

The role of natural disturbance has been comparatively “downplayed in the development of island ecological theory” (Whittaker, 1995) and the few studies that have been conducted have primarily focused on the effects of hurricanes or volcanic activity on oceanic islands (Bush & Whittaker, 1991; Morrison, 2010; Morrison & Spiller, 2008; but see Wardle et al., 1997). Our study is the first to test the pyrodiversity–biodiversity hypothesis on true islands and provides insight into several practical and theoretical dimensions of island disturbance theory. First, pyrodiversity studies on the mainland must contend with the subtle effects of overlapping fire legacies in the surrounding landscape, dubbed the “invisible mosaic” (Brown & York, 2017; Parr & Anderson, 2006). Conducting our study on true islands helps reduce the potential confounding effects from the invisible mosaic since the matrix surrounding our sites is composed of water and not a landscape of overlapping fire legacies that is typical on the mainland.

Second, the notion that the number of species on an island (or in a patch) is determined by a dynamic equilibrium between immigration and extinction rates, as predicted by island biogeography theory (MacArthur & Wilson, 1967), may not apply well to systems that seldom or never reach equilibrium before another disturbance occurs (Whittaker, 1995, 2000). The strong effects of habitat succession (i.e., time since fire) and local habitat heterogeneity on island biotas in our system suggest that wildfire disturbance and recovery, and not island size or isolation, are primary drivers of species turnover among these islands. In contrast, a recent study, which also used fixed‐area sampling plots on lake islands, found a positive relationship between island area and species richness for plants, and both predatory and herbivorous insects (Wang et al., 2024). However, the islands in that lake system differ from ours in several key ways: They lack recurring disturbance, are of similar age, and were created by flooding (Wang et al., 2024). Furthermore, our findings, along with previous studies conducted on these islands (Bell et al., 2017a), demonstrate that the scale of isolation in our island system (i.e., up to 7.9 km from mainland, Appendix S1: Table S1) does not significantly limit immigration rates, even on the most remote islands. As such, application of concepts originally developed at the scale of oceanic islands (i.e., island biogeography theory) do not appear to hold in this or other boreal island systems (Jonsson et al., 2009; Wardle et al., 1997). These findings also echo concerns shared by others about the broad application of these concepts to conservation planning on the mainland (Fahrig, 2020; Norton et al., 2000; Riva & Fahrig, 2022; Wintle et al., 2019).

Third, the recent analysis of fire regime changes in Canada by Hanes et al. (2019) showed that for the Lake Athabasca fire regime zone, whose boundary occurs just north of our study site, annual area burned has increased by >7000 ha year^−1^ between 1959 and 2015. Although this region has a low human population density and suppression of wildfires is “relaxed” in comparison to other regions (see Zahara, 2020), this is the fastest rate of increase in annual area burned of any fire regime zone in Canada (Hanes et al., 2019). Increased fire activity in this region could alter the composition of the patch mosaic, potentially putting unburnt habitats in this region such as old growth forest at risk (Erni et al., 2017). Our study of lake islands suggests that, under a “loss of old growth” scenario, maintaining even small, isolated patches of old growth forest on the mainland would conserve important temporal components of biodiversity, including alpha and beta diversity of beetles, plants, and birds. Furthermore, fire managers and conservationists seeking to protect old growth habitats in this region could make use of natural fire refugia, including those created by large lakes in the region (Nielsen et al., 2016).

We sampled each island in only a single season, but not all in the same year, and therefore assumed that diversity in our island plots was consistent across years. This arose mainly from opportunistic sampling of two recently burnt islands (HF and MI) that ignited while our study was already underway. Furthermore, because of the way mainland wildfires interact with our island system (Nielsen et al., 2016), availability of islands that were both highly isolated and recently burnt was limited, leading to potentially confounding effects of time since fire on richness‐isolation relationships for plants (see Appendix S1: Results). However, this limitation also demonstrates that ignoring key habitat characteristics, such as time since fire, can confound observed relationships between richness and patch size, connectivity, and habitat amount. We also observed a negative relationship between habitat amount and beetle richness even after accounting for the effects of time since fire (Appendix S1: Results), which is at odds with predictions of the habitat amount hypothesis. This finding suggests that additional habitat variables not included in our hypothesis‐based models may also influence beetle richness patterns in our study system.

## CONCLUSION

Our study showed that wildfires and their resulting pyrodiversity on islands was a more accurate predictor of island diversity patterns than was either the size of the islands or their proximity to a larger species pool (i.e., the mainland). These findings underscore the often‐neglected role of disturbance in the development of island theory (Whittaker, 1995) and the risk of improperly applying habitat patch concepts to the challenges presented by changing fire regimes on the mainland. Our study, together with others (Wintle et al., 2019), emphasizes the importance of habitat heterogeneity in sustaining biodiversity, the value of small patches, and the need to consider more deterministic aspects (i.e., habitat type, time since disturbance) of mainland patches, beyond merely their size and connectivity. Whereas many species flourish in the aftermath of wildfires (e.g., pyrophilic insects), predicted increases in fire frequency and extreme weather events (Wang et al., 2015, 2017) indicate that scenarios involving loss of old‐growth habitats may become more common (Erni et al., 2017). Conserving even small patches of old growth forest, through use of natural fire refugia (Nielsen et al., 2016), will aid in preserving vital components of temporal pyrodiversity, and thereby biodiversity.

## AUTHOR CONTRIBUTIONS

*Conceptualization*: Aaron J. Bell, Iain D. Phillips, David A. Wardle. *Methodology*: Aaron J. Bell, David A. Wardle, Steven L. Van Wilgenburg, Colin P. Laroque, Stephen M. A. Paterson. *Validation*: Aaron J. Bell, Steven L. Van Wilgenburg. *Formal analysis*: Aaron J. Bell, Steven L. Van Wilgenburg. *Investigation*: Aaron J. Bell, Iain D. Phillips, Stephen M. A. Paterson. *Resources*: Aaron J. Bell, Iain D. Phillips, Stephen M. A. Paterson, Steven L. Van Wilgenburg, Colin P. Laroque. *Data curation*: Aaron J. Bell. *Writing—original draft preparation*: Aaron J. Bell. *Writing—review and editing*: Aaron J. Bell, Iain D. Phillips, David A. Wardle, Steven L. Van Wilgenburg, Colin P. Laroque, Stephen M. A. Paterson. *Visualization*: Aaron J. Bell. *Supervision*: Iain D. Phillips, David A. Wardle, Colin P. Laroque. *Project administration*: Aaron J. Bell. *Funding acquisition*: Aaron J. Bell, Iain D. Phillips.

## CONFLICT OF INTEREST STATEMENT

The authors declare no conflicts of interest.

## Supporting information


Appendix S1.

## Data Availability

Data (Bell & Van Wilgenburg, 2026) are available in Dryad at https://doi.org/10.5061/dryad.tdz08kq78.
