# Supplementary material for: Pyrodiversity of boreal lake islands begets biodiversity of beetles, plants, and birds
Source: Ecol Appl. 2026 Mar 30;36(2):e70218. doi: 10.1002/eap.70218 (PMC13035461; doi:10.1002/eap.70218)
Supplement: Supplementary file 1 — Appendix S1. [file EAP-36-e70218-s001.pdf]

Pyrodiversity of boreal lake islands begets biodiversity of beetles, plants, and birds

Aaron J. Bell, Stephen M. A. Paterson, Steven L. Van Wilgenburg, Colin P. Laroque, David A. Wardle, and Iain D. Phillips

Ecological Applications

Appendix S1

Methods

Specimens from traps collected in the field were stored in 70% ethanol and sorted under 20X magnification. Adult beetles for most taxa were identified to species using keys for Artematopodidae (Hinson and Buss 2015), Byrrhidae (Johnson 1991), Cantharidae (Pelletier and Hébert 2014), Carabidae (Lindroth 1969), Ciidae (Lawrence 1971, Lopes-Andrade et al. 2016), Chrysomelidae (Smith 1985, Parry 1986, LeSage 1986, Blake 1943), Cleridae (Leavengood 2008), Cryptophagidae (Pelletier and Hébert 2019), Curculionidae (Anderson 1989, Arnett et al. 2002, Warner 1966), Eucinetidae (Majka 2010), Erotylidae (Goodrich and Springer 1999), Histeridae (Bousquet and Laplante 2006), Lampyridae (Majka 2012), Leiodidae (Wheeler 1979, Hatch 1933, Baranowski 1993), Melandryidae (Mank 1939, Pollock 2002), Mycetophagidae (Parsons 1975), Peltidae and Thymalidae (Barron 1971, 1996; Kolibáč 2013), Ptiliidae (Dybas 1966), Ptinidae (White 1966), Scolytinae (Bright 1987), Scirtidae (under ‘Cyphonidae’, Hatch 1962), Sphindidae (Casey 1898), Staphylinidae (Smetana 1971, Herman 1975, Assing and Wunderle 1995, Arnett and Thomas 2000), Tenebrionidae (Aalbu et al. 2002, Peck and Thomas 1998, Gardiner and Pollock 2015), and Tetratomidae (Horn 1888). Species from the subfamily Aleocharinae (Coleoptera: Staphylinidae) could not be identified to genus and species given the time and resource constraints of our study and were thus removed from the analysis. The genera *Acrotrichis* and *Ptenidium* (Ptiliidae), *Baeocera*, *Bisnius*, *Gabrius*, *Lathrobium*, *Megarthrus*, *Paederus*, and *Stenus* (Staphylinidae), *Corticaria* (Latridiidae), and *Pityopthorus* (Curculionidae)

were included in the analysis at the genus-level. Voucher specimens are deposited in the Troutreach Saskatchewan collection, Saskatoon, Saskatchewan, Canada. Body size information for beetles was obtained from the literature and BugGuide (http://bugguide.net), and an average of minimum and maximum reported size values (in mm, to the nearest 0.1) was used in our multi-species occupancy models (see https://doi.org/10.5061/dryad.tdz08kq78, Beetle_species_list.xlsx).

Plants were identified using the keys developed for western Canada (Johnson et al. 1995) and Saskatchewan (Leighton 2012, Leighton and Harms, 2014).

Results

To explore potential confounding effects of time since fire on plant richness relationships, we first fitted a linear model of time since fire to plant richness using a Poisson error distribution (n = 41, *P* = <0.001, IRR =0.68 [CI = 0.61 – 0.74], D^2: 0.81). We saved the residuals from that plant richness model as a metric of whether plant species richness was greater (positive residuals) or lower (negative residuals) than predicted via the relationships with time since fire. We then assessed residual plant species richness in relation to island area, isolation index, and habitat amount with linear regression and a Gaussian error distribution. Using this approach, we found no relationship between the unexplained variation in plant richness and island area (n = 41, *P* = 0.122, IRR = 0.52 [CI = -0.14 – 1.17]), isolation (*P* = 0.997, IRR = 0 [CI = -0.60 – 0.60]), or habitat amount (n = 41, *P* = 0.979, IRR = 0.01 [CI = -0.60 – 0.62]), suggesting that the relationship between plant richness and these spatial variables was instead due to low plant richness in older island forests that also tend to be more isolated in our lake-island system

(Nielsen et al. 2016). We also performed a similar analysis of residuals to examine why the relationship between beetle richness and habitat amount was negative (i.e., increasing richness with decreasing habitat amount). However, when the effects of time since fire on beetle richness were accounted for (using either a linear or quadratic time since fire model), the effect of isolation index on beetle richness varied between significant (null-hypothesis) and non-significant (information-theoretic), depending on which statistical inference was used (n = 42, P = 0.025, IRR = 0.61 [CI = 0.10 – 1.12]). In contrast, the significant negative effects of habitat amount on beetle richness persisted even after accounting for time since fire (n = 42, P = 0.005, IRR = -0.72 [CI = -1.22 – -0.22]).

Table S1. Information on island size (area) and isolation (distance to mainland), time since fire (TSF, in years), source of fire history information, and species richness (*S*) of beetles, plants, and birds on each island, with estimated species richness included in parentheses.

Island	Area (ha)	Distance to ML (km)	TSF	Source	*Sbeetles*	*Splants*	*Sbirds*
BB	6.3	0.35	5	Landsat	75 (96)	15	10 (12)
BC	23.3	1.76	215	Dendro	78 (101)	4	10 (11)
BS	350.4	0.27	75	Dendro	73 (95)	n/a	9 (11)
BT	1.8	0.69	5	Landsat	89 (114)	14	9 (10)
CC	21.1	0.14	176	Dendro	90 (115)	9	16 (18)
CD	3.2	3.87	179	Dendro	89 (115)	5	7 (8)
CG	1.6	0.24	113	Dendro	97 (125)	24	18 (21)
DF	38.3	0.34	5	Landsat	89 (114)	22	10 (12)
DN	29.1	0.11	14	Landsat	73 (94)	29	12 (14)
DS	101.0	0.21	180	Dendro	75 (97)	7	17 (19)
EB	13.8	0.27	5	Landsat	87 (112)	16	13 (15)
EI	2.9	0.15	76	Dendro	105 (136)	23	10 (12)
EL	10.0	6.94	115	Dendro	95 (123)	5	8 (9)
FD	35.7	0.07	14	Landsat	69 (90)	42	13 (15)
HF	11.8	0.88	1	Landsat	93 (119)	13	9 (10)
HI	35.0	0.02	39	Landsat + maps	66 (86)	30	10 (11)
HU	1.2	3.15	10	Landsat	99 (128)	26	4 (5)
IL	19.8	0.45	25	Landsat	76 (99)	24	13 (15)
IS	5.9	0.33	25	Landsat	83 (108)	32	6 (7)
JO	130.2	6.58	111	Dendro	105 (136)	16	9 (10)
KA	3.9	2.10	125	Dendro	91 (119)	21	9 (10)
KC	3.4	1.23	69	Dendro	69 (90)	22	7 (8)
KP	2.7	0.14	39	Landsat + maps	71 (92)	13	9 (10)
KR	4.5	0.07	39	Landsat + maps	65 (85)	4	10 (12)
LQ	26.9	7.03	164	Dendro	100 (129)	5	10 (11)
MI	1.2	2.50	1	Landsat	128 (162)	19	11 (13)
MN	1.7	0.15	38	Landsat + maps	67 (87)	19	12 (13)

MT	7.5	0.38	134	Dendro	85 (109)	7	10 (12)
NH	11.3	0.23	25	Landsat	71 (93)	24	14 (16)
NV	16.8	1.84	129	Dendro	89 (114)	6	8 (9)
NW	53.9	1.72	216	Dendro	105 (137)	18	10 (12)
OS	4.0	0.16	5	Landsat	62 (80)	12	5 (6)
PP	9.4	0.15	14	Landsat	73 (95)	22	14 (16)
PR	1.0	0.17	14	Landsat	73 (94)	14	13 (15)
QC	10.5	0.04	82	Dendro	67 (87)	5	7 (8)
SF	131.0	0.18	14	Landsat	76 (99)	34	16 (18)
SG	47.7	7.90	231	Dendro	62 (80)	4	12 (14)
SK	4.8	0.06	14	Landsat	71 (92)	28	13 (15)
SR	1.9	0.54	83	Dendro	88 (114)	6	9 (10)
TB	19.5	6.63	82	Dendro	82 (107)	12	7 (8)
WD	52.7	0.08	5	Landsat	70 (90)	18	6 (7)
WF	49.8	0.64	5	Landsat	79 (102)	28	6 (7)

[1]Time since fire was calculated to a fixed-year (2020) based on the age of the oldest tree at each site to account for minor variation in the year beetles (2020, 2022), plants (2021), and birds (2019 – 2022) were sampled.

[2]Species richness of plants on island BS was not recorded because our study site on this island burned before the plant survey could be conducted.

Table S2. List of candidate models evaluating species-level detection covariates in our multi-species occupancy analysis for beetles and birds, with island as our only site-level occupancy covariate.

Taxa	Model Structure	K	AIC	Δ_i
Beetles				
	ψ(island), *p*(feeding guild[1], body size[2])	50	35597.86	0
	ψ(island), *p*(feeding guild)	49	35611.38	13.52
	ψ(island), *p*(body size)	44	36395.29	797.43
	ψ(island), *p*(.)	43	36426.16	828.30
Birds				
	ψ(island), *p*(log(mass)[3], MaxFreqkHz[4], migration factor[5])	46	5667.94	0
	ψ(island), *p*(migration factor)	44	5674.02	6.08
	ψ(island), *p*(log(mass))	44	5752.72	84.78
	ψ(island), *p*(MaxFreqkHz)	44	5776.45	108.52
	ψ(island), *p*(.)	43	5777.67	109.73

[1]Beetle species were assigned to one of seven feeding guilds: carnivores, fungivores, herbivores, omnivores, palynivores, saprovores, and saproxylics.
[2]Beetle body size was measured from the tip of the mandibles to the apex of the elytra and included as a continuous variable.
[3]Bird body mass (g) was log-transformed for analysis.
[4]Maximum sound frequency (kHz) of bird vocalizations divided by ten for analysis.
[5]Bird migration strategy (migrants vs residents).

Table S3. List of potential candidate habitat variables included in our Redundancy Analysis of beetle, plant, and bird species matrices.

Variables	Description	Beetles	Plants	Birds
plant.richness	plant species richness	*0.015		0.730
log10area	log10(area+1) of island area	0.080	0.160	0.275
buffer5000	island isolation index (0 – 1)	*0.050	0.150	0.070
time.since.fire	years elapsed since last fire	****0.005**	***0.005**	****0.005**
volume.CWD.without.charcoal	volume of CWD without charcoal	0.075	0.170	0.405
volume.fire-killed.CWD	volume of fire-killed CWD	****0.005**	****0.005**	0.310
total.CWD.volume	total volume of CWD	*0.020		
CWD.dc1	volume of CWD in decay class 1	*0.035		
CWD.dc2	volume of CWD in decay class 2	0.065		
CWD.dc3	volume of CWD in decay class 3	0.130		
CWD.dc4	volume of CWD in decay class 4	0.495		
CWD.dc5	volume of CWD in decay class 5	0.100		
decid.overstory	% cover of deciduous canopy	**0.005	***0.020**	0.055
decid.understory	% cover of deciduous understory	**0.005	****0.005**	****0.005**
conifer.overstory	% cover of coniferous canopy	*0.010	0.640	0.600
conifer.understory	% cover of coniferous understory	0.095	0.055	****0.010**
bird.abundance	total # of bird counts	0.240		
basal.area	cross-sectional area of trees at DBH	**0.010	***0.040**	0.465
insectivore.abund	# of insectivorous birds	0.715		
frugivore.abund	# of frugivorous birds	*0.015	0.625	
beetle.catch.rate	# of indiv. divided by trap-days			0.335

Note: Bolded *P*-values indicate variables that were included in the top model for each taxon, as chosen by a step function model selection. Cells without values indicate candidate variables that were excluded in the analysis for that taxon. Abbreviations: coarse woody debris (CWD), diameter breast height (DBH).

Table S4. Complete list of 101 plant species sampled on 42 lake islands (1 to 231+ years since fire) in the Lac la Ronge region following the nomenclature of Johnson et al. 1995; Harms and Leighton 2011; Leighton and Harms 2020).

**Order**	**Family**	**Species**
Apiales	Araliaceae	*Aralia nudicaulis*
		Aralia hispida
Asparagales	Asparagaceae	*Maianthemum canadense*
	Orchidaceae	*Cypripedum acaule*
		Goodyera repens
Asterales	Asteraceae	*Achillea millefolium*
		Achillea sibirica
		Cirsium arvense
		Conyza canadensiss
		Erigeron glabellus palmatus
		Petasites frigidus
		Symphyotrichum ciliolatus
		Taraxacum officinale
Boraginales	Boraginaceae	*Mertensia paniculata*
Caryophyllales	Caryophyllaceae	*Arenaria lateriflora*
		Stellaria calycantha
		Stellaria longifolia
	Polygonaceae	*Fallopia cilinodis*
Cornales	Cornaceae	*Cornus canadensis*
		Cornus stolonifera
Cupressales	Cupressaceae	*Juniperus communis*
Dipsacales	Adoxaceae	*Sambucus racemosa*
		Viburnum edule
	Caprifoliaceae	*Linnaea borealis*
		Lonicera dioica
Equisetales	Equisetaceae	*Equisetum arvense*
		Equisetum sylvaticum
Ericales	Balsaminaceae	*Impatiens capensis*
	Ericaceae	*Arctostaphylos uva-ursi*
		Chimaphila umbellata
		Orthilia secunda
		Pyrola asarifolia
		Pyrola secunda
		Pyrola virens
		Rhododendron groenlandicum
		Vaccinium myrtilloides
		Vaccinium oxycoccos

		Vaccinium vitis-idaea
	Primulaceae	*Trientalis borealis*
Fabales	Fabaceae	*Lathyrus ochroleucus*
		Lathyrus venosus
		Vicia americana
Fagales	Betulaceae	*Alnus viridis*
		Betula papyrifera
Geraniales	Geraniaceae	*Geranium bicknellii*
Lamiales	Lamiaceae	*Lycopus uniflorus*
	Orobanchaceae	*Melampyrum lineare*
Lycopodiales	Lycopodiaceae	*Lycopodium complanatum*
		Lycopodium obscurum
Malpighiales	Salicaceae	*Populus tremuloides*
		Populus balsamifera
		Salix bebbiana
		Salix spp.
	Violaceae	*Viola renifolia*
Myrtales	Onagraceae	*Chamaenerion angustifolium*
		Epilobium ciliatum
		Epilobium palustre
Pinales	Pinaceae	*Abies balsamea*
		Picea glauca
		Picea mariana
		Pinus banksiana
Poales	Cyperaceae	*Carex foenea*
		Carex brunnescens
		Carex deflexa
		Carex disperma
		Carex dewayana
		Carex houghtoniana
		Scirpus atrocinctus
	Poaceae	*Agrostis scabra*
		Calamagrostis canadensis
		Calamagrostis stricta
		Calamagrostis spp
		Festuca saximontana
		Muhlenbergia spp
		Poa palustris
		Poaceae sp.1
		Poaceae sp.2
		Poaceae sp.3

Polypodiales	Cystopteridaceae	*Gymnocarpium dryopteris*
	Dryopteridaceae	*Dryopteris austriaca*
	Polypodiaceae	*Polypodium virginianum*
Ranunculales	Ranunculaceae	*Actaea rubra*
		Anemone canadensis
	Papaveraceae	*Corydalis sempervirens*
Rosales	Elaeagnaceae	*Shepherdia canadensis*
	Rosaceae	*Amelanchier alnifolia*
		Fragaria spp.
		Potentilla norvegica
		Prunus pennsylvanica
		Rosa acicularis
		Rubus idaeus
		Rubus pubescens
		Sorbus scopulina
	Urticaceae	*Urtica dioica*
Santalales	Santalalaceae	*Geocaulon lividum*
Saxifragales	Grossulariaceae	*Ribes glandulosum*
		Ribes lacustris
		Ribes oxycanthoides
		Ribes triste
	Saxifragaceae	*Mitella nuda*
		Saxifraga tricuspidata

Table S5. Complete list of 54 bird species sampled on 42 lake islands (1 to 231+ years since fire in the Lac la Ronge region.

Family	Species
Bombycillidae	*Bombycilla cedrorum* Vieillot, 1808
Caprimulgidae	*Chordeiles minor* Forstery, 1771
Certhiidae	*Certhia americana* Bonaparte, 1838
Fringillidae	*Loxia curvirostra* Linnaeus, 1758
	Loxia leucoptera Gmelink, 1789
	Spinus pinus Wilson, 1810
Hirundinidae	*Tachycineta bicolor* Vieillot, 1808
Icteridae	*Agelaius phoeniceus* Linnaeus, 1766
	Euphagus carolinus Muller, 1776
	Quiscalus quiscula Linnaeus, 1758
Paridae	*Poecile hudsonicus* Forster, 1772
Parulidae	*Cardellina pusilla* Wilson, 1811
	Geothlypis philadelphia Wilson, 1810
	Leiothlypis celata Say, 1822
	Leiothlypis peregrina Wilson, 1811
	Mniotilta varia Linnaeus, 1766
	Parkesia noveboracensis Gmelin, 1789
	Seiurus aurocapilla Linnaeus, 1766
	Setophaga castanea Wilson, 1810
	Setophaga coronata Linnaeus, 1766
	Setophaga magnolia Wilson, 1811
	Setophaga petechia Linnaeus, 1766
	Setophaga ruticilla Linnaeus, 1758
Passerellidae	*Junco hyemalis* Linnaeus, 1758
	Spizella passerina Bechstein, 1798
	Melospiza georgiana Latham, 1790
	Melospiza lincolnii Audubon, 1834
	Melospiza melodia Wilson, 1810
	Passerculus sandwichensis Gmelin, 1789
	Zonotrichia albicollis Gmelin, 1789
Phasianidae	*Bonasa umbellus* Linnaeus, 1766
	Canachites canadensis Linnaeus, 1758
Picidae	*Colaptes auratus* Linnaeus, 1758
	Dryobates pubescens Linnaeus, 1766
	Dryocopus pileatus Linnaeus, 1758
	Picoides arcticus Swainson, 1832
	Picoides dorsalis Baird, 1858
	Sphyrapicus varius Linnaeus, 1766

Regulidae	*Corthylio calendula* Linnaeus, 1766
	Regulus satrapa Lichtenstein, 1823
Scolopacidae	*Actitis macularius* Linnaeus, 1766
	Gallinago delicata Ord, 1825
	Tringa melanoleuca Gmelin, 1789
	Tringa solitaria Wilson, 1813
Sittidae	*Sitta canadensis* Linnaeus, 1766
Troglodytidae	*Troglodytes aedon* Vieillot, 1809
	Troglodytes hiemalis Vieillot, 1819
Turdidae	*Catharus guttatus* Pallas, 1811
	Catharus ustulatus Nuttall, 1840
	Turdus migratorius Linnaeus, 1766
Tyrannidae	*Empidonax alnorum* Brewster, 1895
	Empidonax minimus Baird, 1843
Vireonidae	*Vireo olivaceus* Linnaeus, 1766
	Vireo philadelphicus Cassin, 1851

Table S6. Incident rate ratios (IRR) and confidence intervals (CI) for Poisson generalized linear regression models explaining factors affecting species richness of beetles, plants, and birds on the islands in our study.

		Beetles			Plants			Birds		
Model	Variable	IRR	CI	*P*	IRR	CI	*P*	IRR	CI	*P*
Pyrodiversity	intercept	0.13	0.12 - 0.14	<0.001	24.37	21.27 - 27.78	<0.001	11.71	10.22 - 13.33	<0.001
(n = 19 islands)	sd.fire.severity	1.02	0.97 - 1.07	0.433	1.24	1.11 - 1.39	<0.001	1.22	1.07 - 1.39	0.004
	time.since.fire	0.90	0.85 - 0.94	<0.001	1.26	1.12 - 1.43	<0.001	1.11	0.97 - 1.26	0.132
	time.since.fire^2	1.05	1.00 - 1.10	0.066	0.90	0.81 - 1.00	0.046			
	sd.fire.severity*time.since.fire				0.85	0.74 - 0.98	0.020			
Island Biogeography	intercept	0.13	0.13 - 0.14	<0.001	16.71	15.48 - 18.01	<0.001	11.73	10.72 - 12.80	<0.001
(n = 42 islands)	$\log_{10}$(area+1)	0.99	0.96 - 1.02	0.517	1.12	1.03 - 1.22	0.009	1.06	0.97 - 1.16	0.186
	isolation index	1.07	1.04 - 1.10	<0.001	0.80	0.74 - 0.87	<0.001	0.92	0.83 - 1.00	0.058
Habitat Amount	intercept	0.13	0.13 - 0.14	<0.001	16.52	15.29 - 17.81	<0.001	11.74	10.74 - 12.81	<0.001
(n = 42 islands)	%land.buffer	0.93	0.90 - 0.96	<0.001	1.28	1.18 - 1.38	<0.001	1.09	1.00 - 1.19	0.061

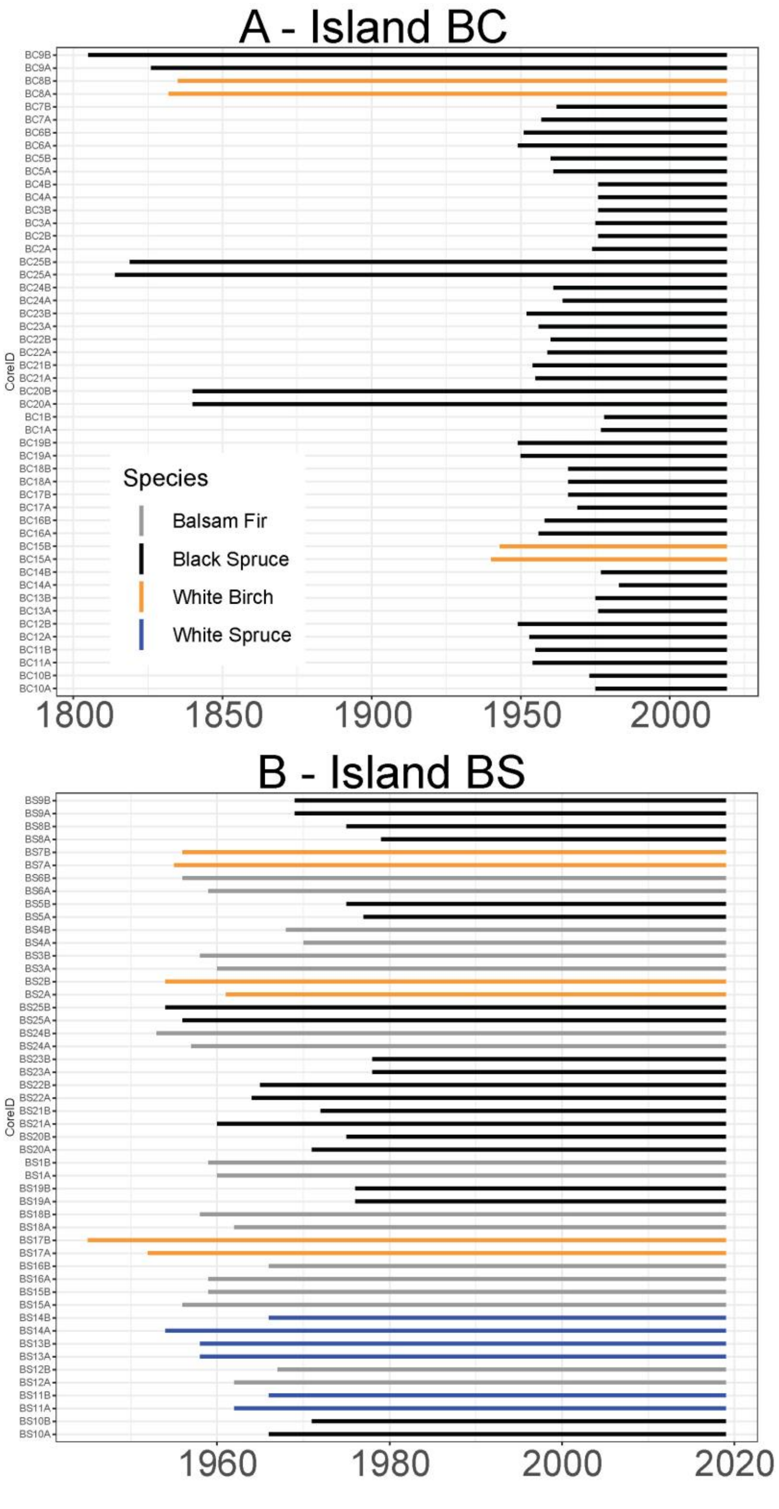

A - Island BC
B - Island BS
CoreID
Species
Balsam Fir
Black Spruce
White Birch
White Spruce
1800
1850
1900
1950
2000
1960
1980
2000
2020

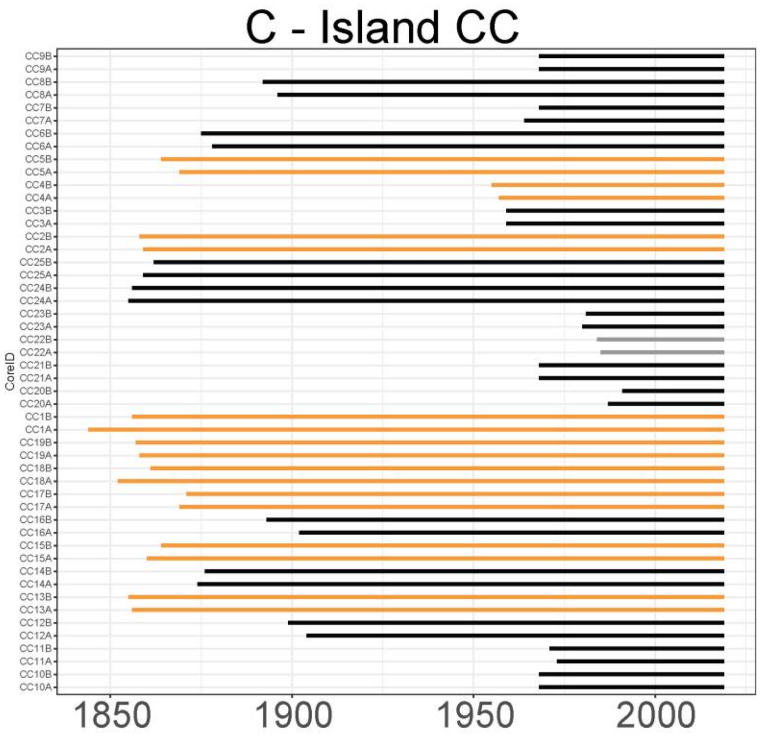
C - Island CC
CoreID
CC9B
CC9A
CC8B
CC8A
CC7B
CC7A
CC6B
CC6A
CC5B
CC5A
CC4B
CC4A
CC3B
CC3A
CC2B
CC2A
CC25B
CC25A
CC24B
CC24A
CC23B
CC23A
CC22B
CC22A
CC21B
CC21A
CC20B
CC20A
CC1B
CC1A
CC19B
CC19A
CC18B
CC18A
CC17B
CC17A
CC16B
CC16A
CC15B
CC15A
CC14B
CC14A
CC13B
CC13A
CC12B
CC12A
CC11B
CC11A
CC10B
CC10A
1850
1900
1950
2000

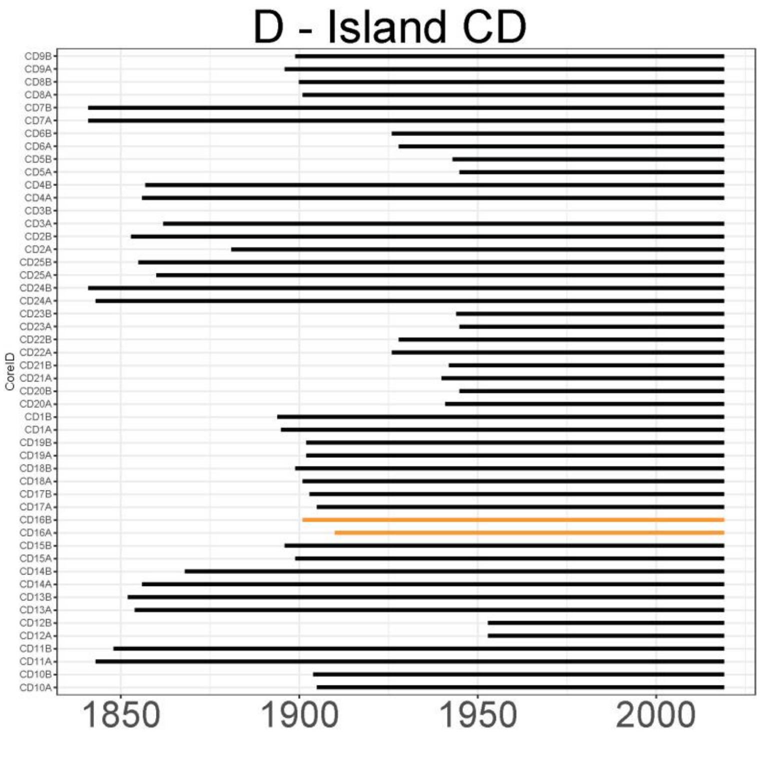
D - Island CD
CoreID
CD9B
CD9A
CD8B
CD8A
CD7B
CD7A
CD6B
CD6A
CD5B
CD5A
CD4B
CD4A
CD3B
CD3A
CD2B
CD2A
CD25B
CD25A
CD24B
CD24A
CD23B
CD23A
CD22B
CD22A
CD21B
CD21A
CD20B
CD20A
CD1B
CD1A
CD19B
CD19A
CD18B
CD18A
CD17B
CD17A
CD16B
CD16A
CD15B
CD15A
CD14B
CD14A
CD13B
CD13A
CD12B
CD12A
CD11B
CD11A
CD10B
CD10A
1850
1900
1950
2000

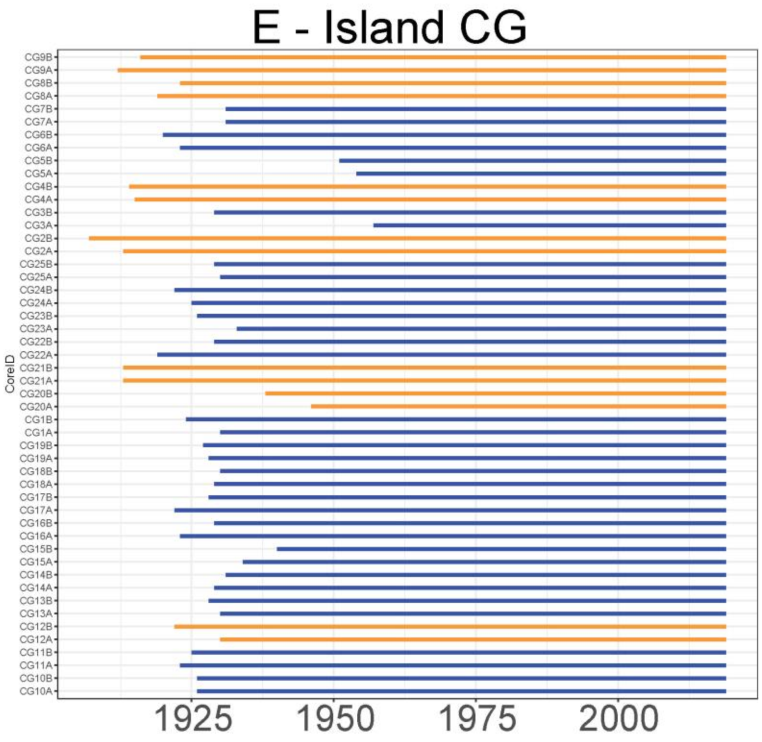
E - Island CG
CoreID
CG9B
CG9A
CG8B
CG8A
CG7B
CG7A
CG6B
CG6A
CG5B
CG5A
CG4B
CG4A
CG3B
CG3A
CG2B
CG2A
CG25B
CG25A
CG24B
CG24A
CG23B
CG23A
CG22B
CG22A
CG21B
CG21A
CG20B
CG20A
CG1B
CG1A
CG19B
CG19A
CG18B
CG18A
CG17B
CG17A
CG16B
CG16A
CG15B
CG15A
CG14B
CG14A
CG13B
CG13A
CG12B
CG12A
CG11B
CG11A
CG10B
CG10A
1925
1950
1975
2000

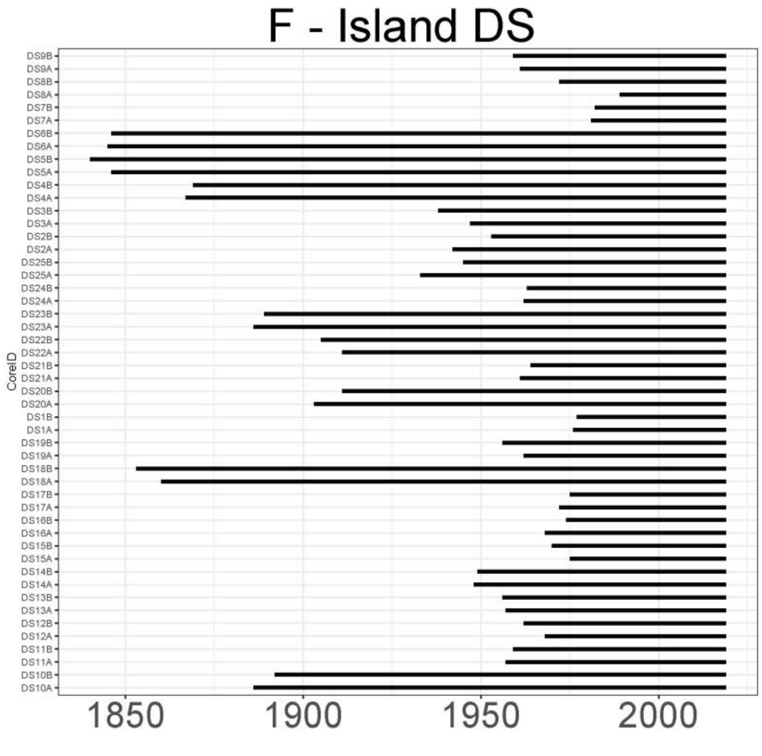
F - Island DS
CoreID
DS9B
DS9A
DS8B
DS8A
DS7B
DS7A
DS6B
DS6A
DS5B
DS5A
DS4B
DS4A
DS3B
DS3A
DS2B
DS2A
DS25B
DS25A
DS24B
DS24A
DS23B
DS23A
DS22B
DS22A
DS21B
DS21A
DS20B
DS20A
DS1B
DS1A
DS19B
DS19A
DS18B
DS18A
DS17B
DS17A
DS16B
DS16A
DS15B
DS15A
DS14B
DS14A
DS13B
DS13A
DS12B
DS12A
DS11B
DS11A
DS10B
DS10A
1850
1900
1950
2000

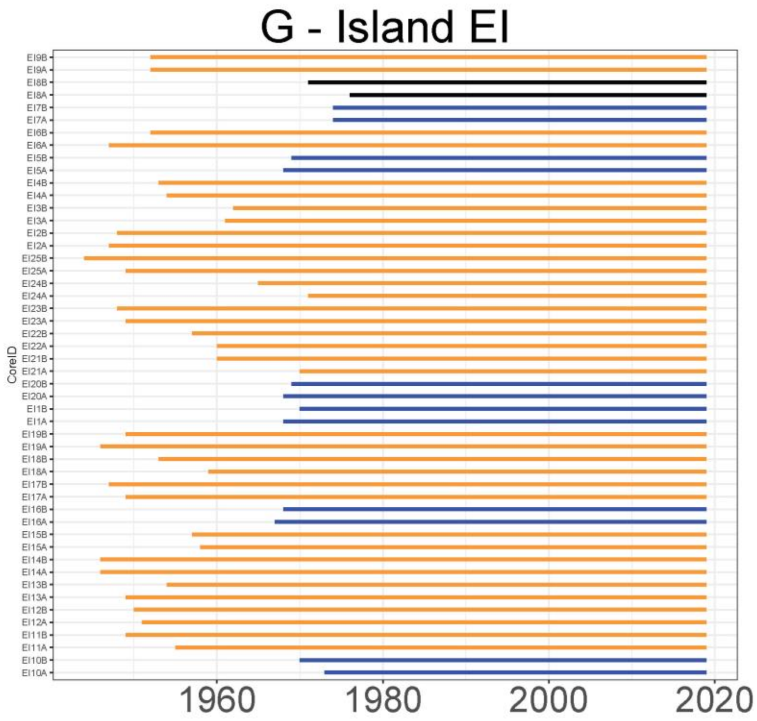
G - Island EI
CoreID
EI9B
EI9A
EI8B
EI8A
EI7B
EI7A
EI6B
EI6A
EI5B
EI5A
EI4B
EI4A
EI3B
EI3A
EI2B
EI2A
EI25B
EI25A
EI24B
EI24A
EI23B
EI23A
EI22B
EI22A
EI21B
EI21A
EI20B
EI20A
EI1B
EI1A
EI19B
EI19A
EI18B
EI18A
EI17B
EI17A
EI16B
EI16A
EI15B
EI15A
EI14B
EI14A
EI13B
EI13A
EI12B
EI12A
EI11B
EI11A
EI10B
EI10A
1960
1980
2000
2020

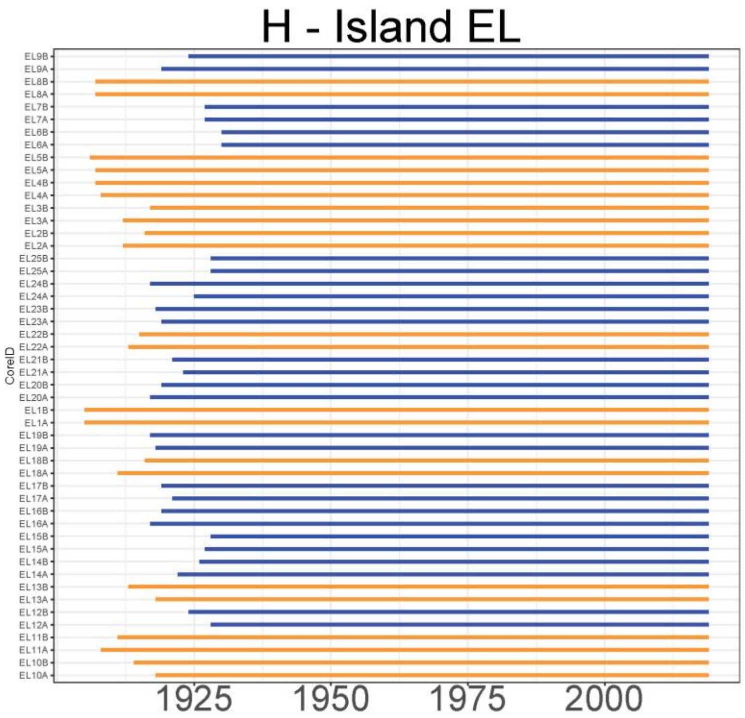
H - Island EL
CoreID
EL9B
EL9A
EL8B
EL8A
EL7B
EL7A
EL6B
EL6A
EL5B
EL5A
EL4B
EL4A
EL3B
EL3A
EL2B
EL2A
EL25B
EL25A
EL24B
EL24A
EL23B
EL23A
EL22B
EL22A
EL21B
EL21A
EL20B
EL20A
EL1B
EL1A
EL19B
EL19A
EL18B
EL18A
EL17B
EL17A
EL16B
EL16A
EL15B
EL15A
EL14B
EL14A
EL13B
EL13A
EL12B
EL12A
EL11B
EL11A
EL10B
EL10A
1925
1950
1975
2000

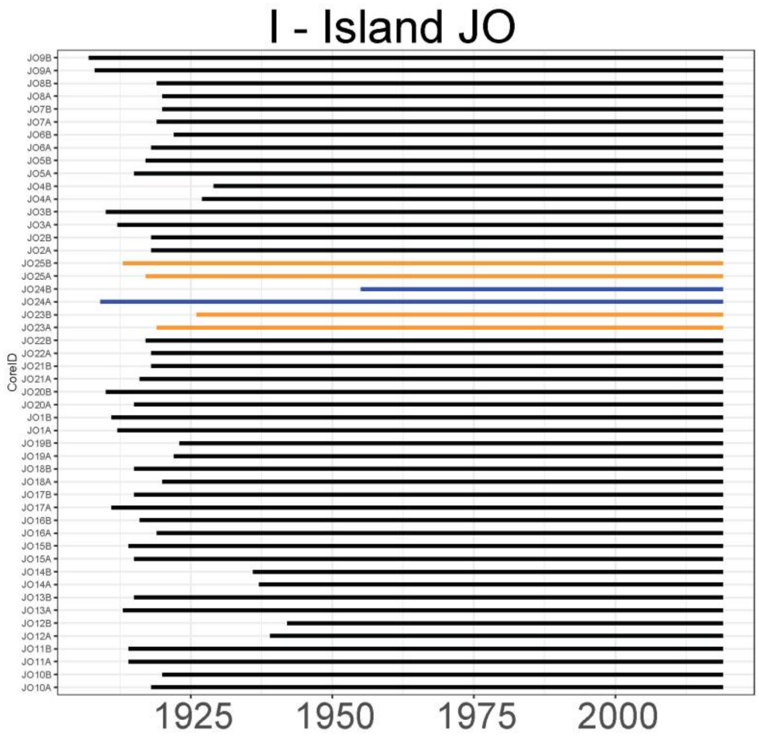
I - Island JO
CoreID
JO9B
JO9A
JO8B
JO8A
JO7B
JO7A
JO6B
JO6A
JO5B
JO5A
JO4B
JO4A
JO3B
JO3A
JO2B
JO2A
JO25B
JO25A
JO24B
JO24A
JO23B
JO23A
JO22B
JO22A
JO21B
JO21A
JO20B
JO20A
JO1B
JO1A
JO19B
JO19A
JO18B
JO18A
JO17B
JO17A
JO16B
JO16A
JO15B
JO15A
JO14B
JO14A
JO13B
JO13A
JO12B
JO12A
JO11B
JO11A
JO10B
JO10A
1925
1950
1975
2000

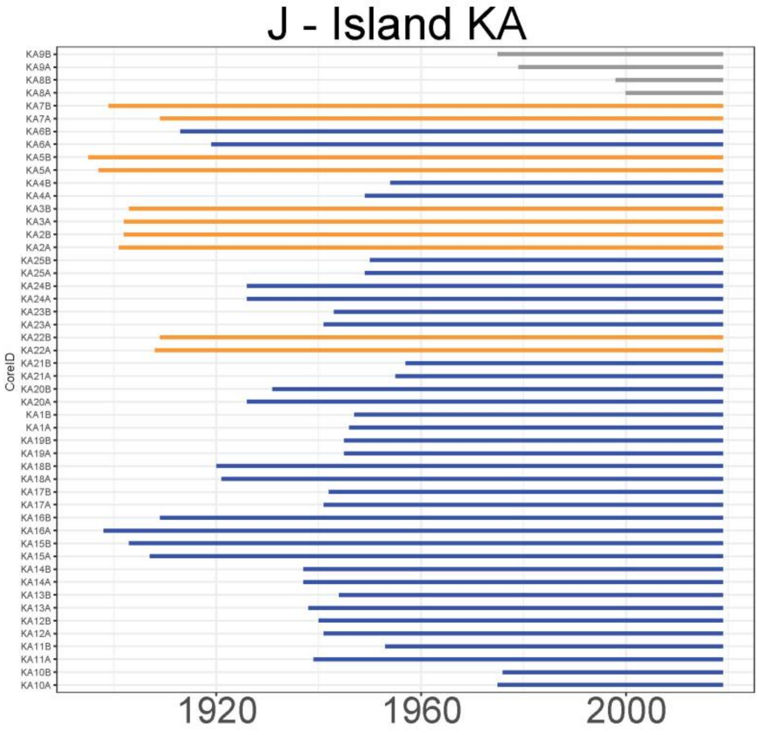
J - Island KA
CoreID
KA9B
KA9A
KA8B
KA8A
KA7B
KA7A
KA6B
KA6A
KA5B
KA5A
KA4B
KA4A
KA3B
KA3A
KA2B
KA2A
KA25B
KA25A
KA24B
KA24A
KA23B
KA23A
KA22B
KA22A
KA21B
KA21A
KA20B
KA20A
KA1B
KA1A
KA19B
KA19A
KA18B
KA18A
KA17B
KA17A
KA16B
KA16A
KA15B
KA15A
KA14B
KA14A
KA13B
KA13A
KA12B
KA12A
KA11B
KA11A
KA10B
KA10A
1920
1960
2000

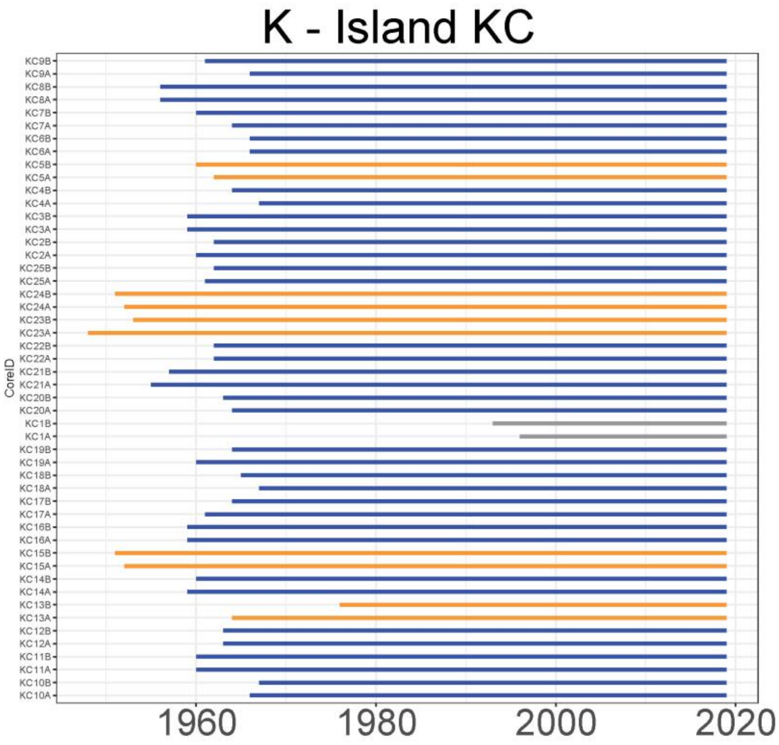

K - Island KC
KC9B
KC9A
KC8B
KC8A
KC7B
KC7A
KC6B
KC6A
KC5B
KC5A
KC4B
KC4A
KC3B
KC3A
KC2B
KC2A
KC25B
KC25A
KC24B
KC24A
KC23B
KC23A
KC22B
KC22A
KC21B
KC21A
KC20B
KC20A
KC1B
KC1A
KC19B
KC19A
KC18B
KC18A
KC17B
KC17A
KC16B
KC16A
KC15B
KC15A
KC14B
KC14A
KC13B
KC13A
KC12B
KC12A
KC11B
KC11A
KC10B
KC10A
CoreID
1960
1980
2000
2020

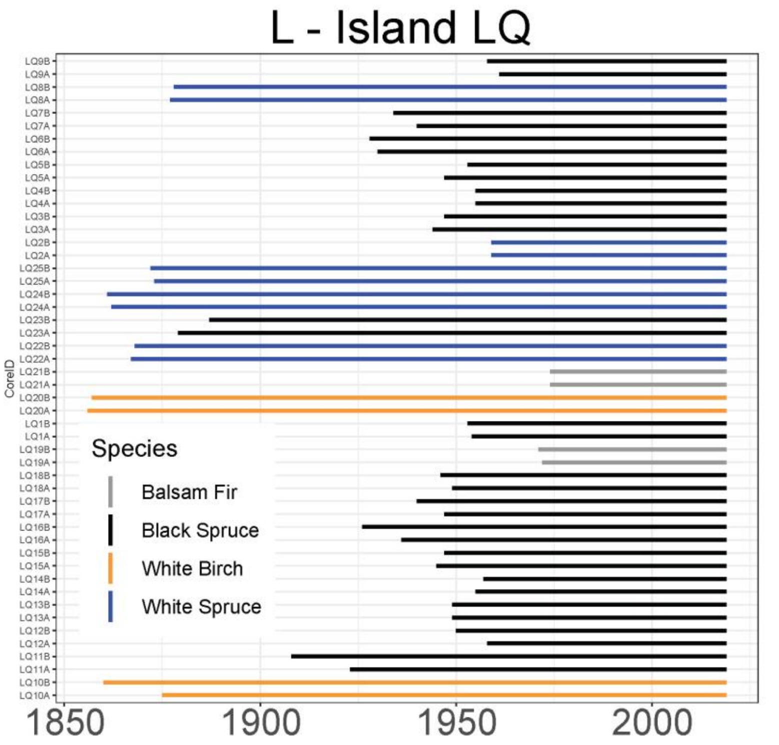

L - Island LQ
LQ9B
LQ9A
LQ8B
LQ8A
LQ7B
LQ7A
LQ6B
LQ6A
LQ5B
LQ5A
LQ4B
LQ4A
LQ3B
LQ3A
LQ2B
LQ2A
LQ25B
LQ25A
LQ24B
LQ24A
LQ23B
LQ23A
LQ22B
LQ22A
LQ21B
LQ21A
LQ20B
LQ20A
LQ1B
LQ1A
LQ19B
LQ19A
LQ18B
LQ18A
LQ17B
LQ17A
LQ16B
LQ16A
LQ15B
LQ15A
LQ14B
LQ14A
LQ13B
LQ13A
LQ12B
LQ12A
LQ11B
LQ11A
LQ10B
LQ10A
CoreID
Species
Balsam Fir
Black Spruce
White Birch
White Spruce
1850
1900
1950
2000

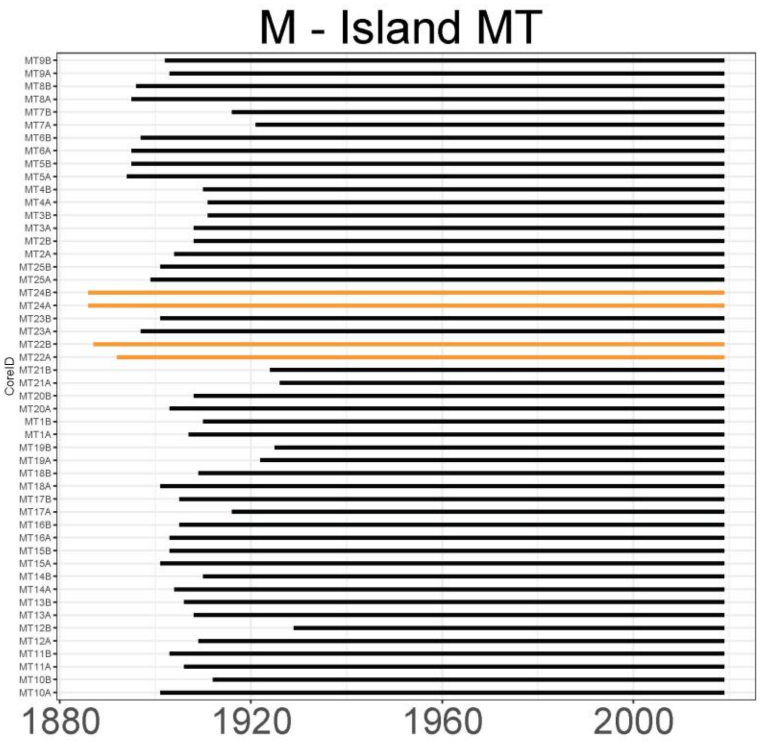
M - Island MT
CoreID
MT9B
MT9A
MT8B
MT8A
MT7B
MT7A
MT6B
MT6A
MT5B
MT5A
MT4B
MT4A
MT3B
MT3A
MT2B
MT2A
MT25B
MT25A
MT24B
MT24A
MT23B
MT23A
MT22B
MT22A
MT21B
MT21A
MT20B
MT20A
MT1B
MT1A
MT19B
MT19A
MT18B
MT18A
MT17B
MT17A
MT16B
MT16A
MT15B
MT15A
MT14B
MT14A
MT13B
MT13A
MT12B
MT12A
MT11B
MT11A
MT10B
MT10A
1880
1920
1960
2000

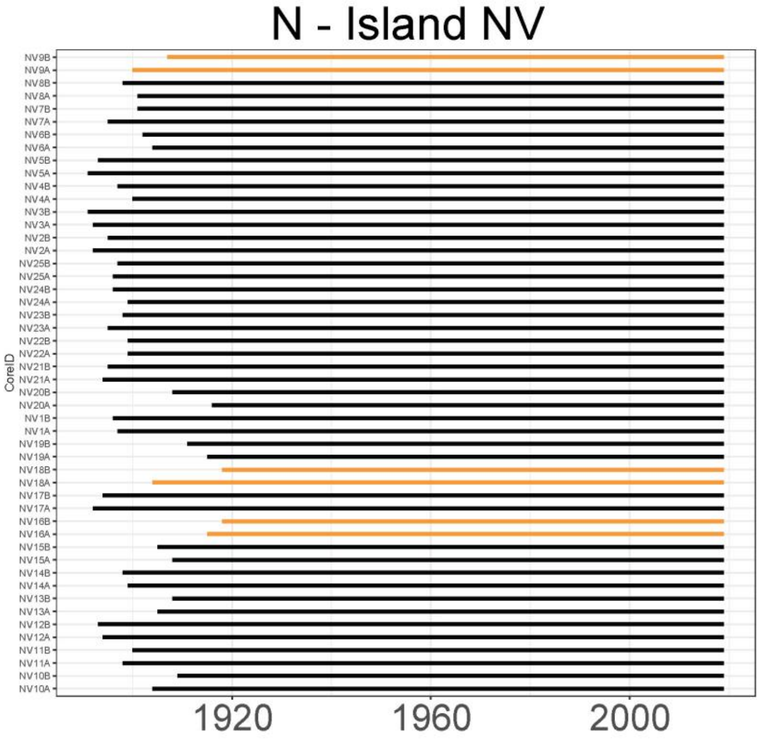
N - Island NV
CoreID
NV9B
NV9A
NV8B
NV8A
NV7B
NV7A
NV6B
NV6A
NV5B
NV5A
NV4B
NV4A
NV3B
NV3A
NV2B
NV2A
NV25B
NV25A
NV24B
NV24A
NV23B
NV23A
NV22B
NV22A
NV21B
NV21A
NV20B
NV20A
NV1B
NV1A
NV19B
NV19A
NV18B
NV18A
NV17B
NV17A
NV16B
NV16A
NV15B
NV15A
NV14B
NV14A
NV13B
NV13A
NV12B
NV12A
NV11B
NV11A
NV10B
NV10A
1920
1960
2000

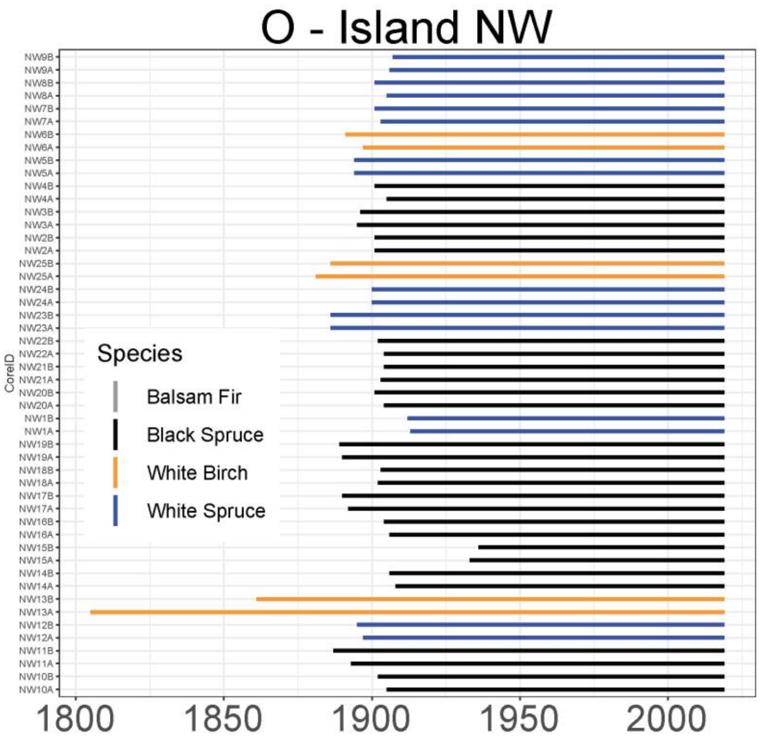
O - Island NW
CoreID
NW9B
NW9A
NW8B
NW8A
NW7B
NW7A
NW6B
NW6A
NW5B
NW5A
NW4B
NW4A
NW3B
NW3A
NW2B
NW2A
NW25B
NW25A
NW24B
NW24A
NW23B
NW23A
NW22B
NW22A
NW21B
NW21A
NW20B
NW20A
NW1B
NW1A
NW19B
NW19A
NW18B
NW18A
NW17B
NW17A
NW16B
NW16A
NW15B
NW15A
NW14B
NW14A
NW13B
NW13A
NW12B
NW12A
NW11B
NW11A
NW10B
NW10A
Species
Balsam Fir
Black Spruce
White Birch
White Spruce
1800
1850
1900
1950
2000

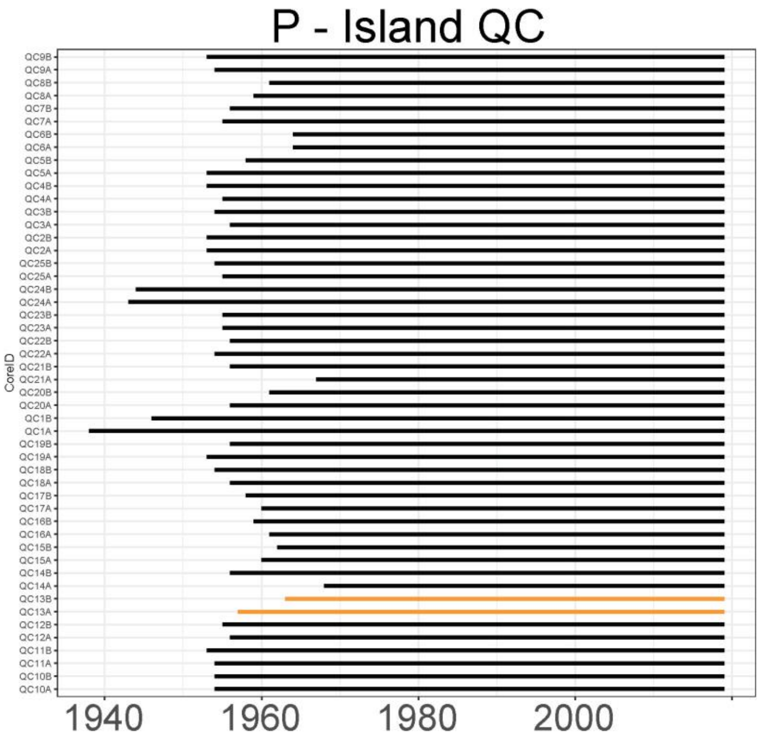
P - Island QC
CoreID
QC9B
QC9A
QC8B
QC8A
QC7B
QC7A
QC6B
QC6A
QC5B
QC5A
QC4B
QC4A
QC3B
QC3A
QC2B
QC2A
QC25B
QC25A
QC24B
QC24A
QC23B
QC23A
QC22B
QC22A
QC21B
QC21A
QC20B
QC20A
QC1B
QC1A
QC19B
QC19A
QC18B
QC18A
QC17B
QC17A
QC16B
QC16A
QC15B
QC15A
QC14B
QC14A
QC13B
QC13A
QC12B
QC12A
QC11B
QC11A
QC10B
QC10A
1940
1960
1980
2000

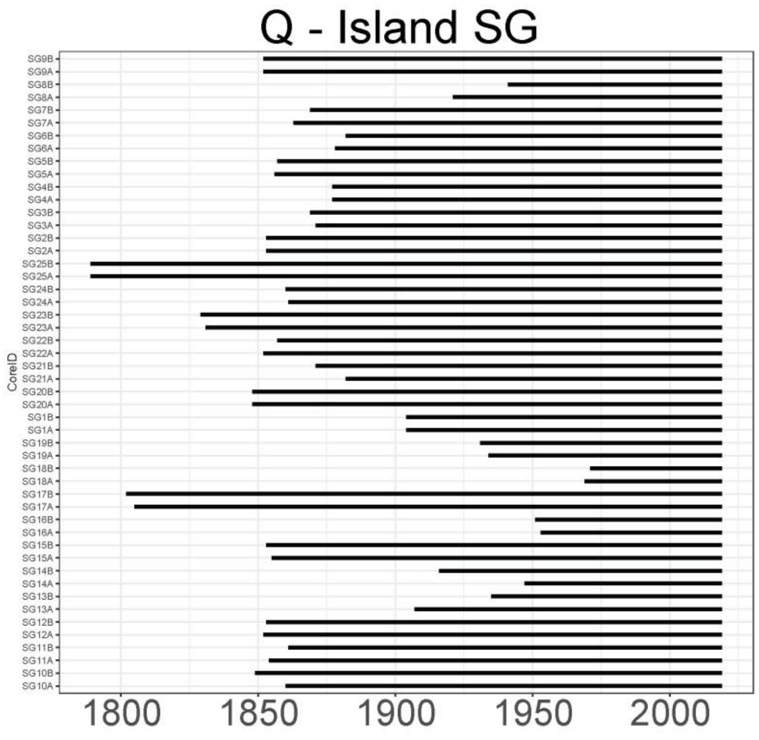
Q - Island SG
CoreID
1800
1850
1900
1950
2000

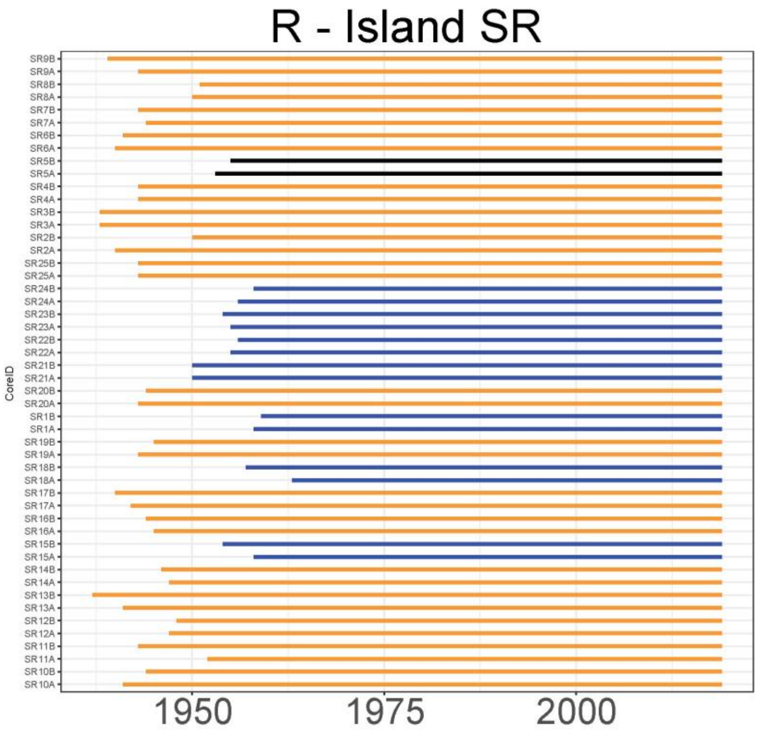
R - Island SR
CoreID
1950
1975
2000

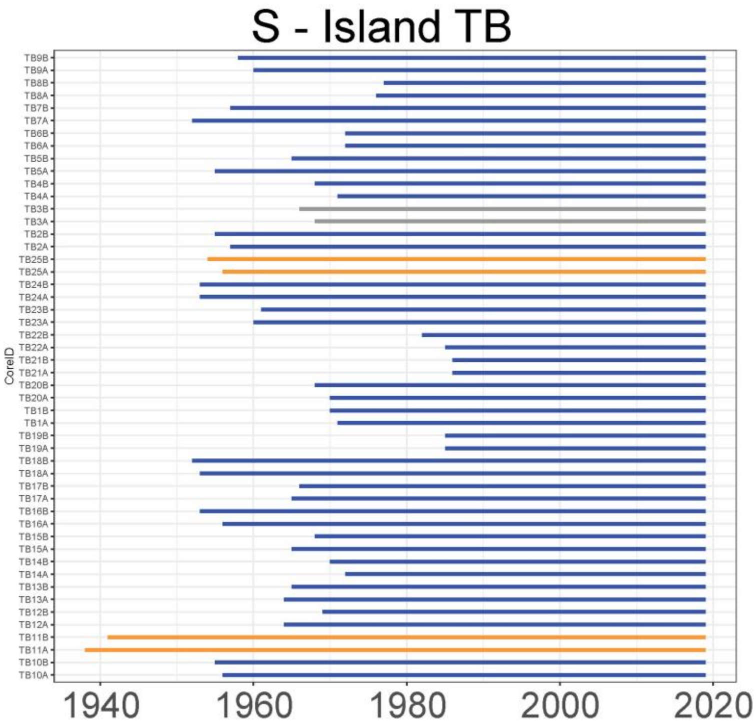

Figure S1. Stand-age reconstruction for islands (n = 19) that burned pre-1981 based on the 25 living trees nearest the center of each island plot: A) island BC, B) island BS, C) island CC, D) island CD, E) island CG, F) island DS, G) island EI, H) island EL, I) island JO, J) island KA, K) island KC, L) island LQ, M) island MT, N) island NV, O) island NW, P) island QC, Q) island SG, R) island SR, S) island TB. Refer to Table S1 for island characteristics.

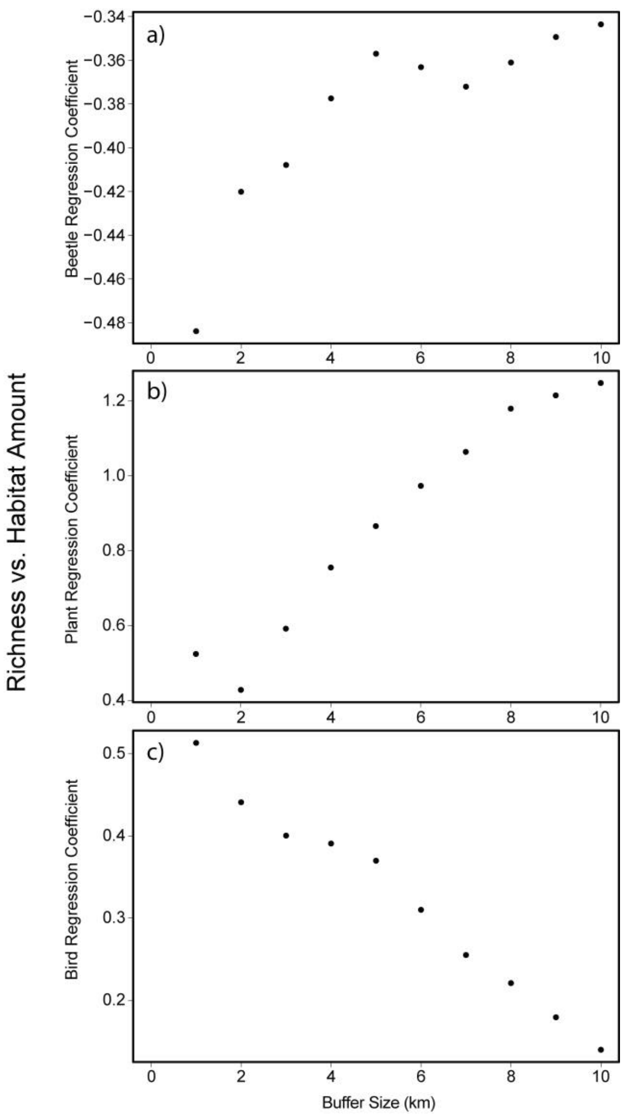

Figure S2. A multi-scale analysis examining the relationship between species richness and habitat amount (i.e., the scale of effect, see Fahrig 2013) at multiple spatial extents (buffer sizes 1 – 10 km) for beetles (a), plants (b), and birds (c). The habitat amount hypothesis was evaluated for each taxon based on the spatial extent with the largest regression coefficient: 10 km for plants, 1 km for beetles and birds.

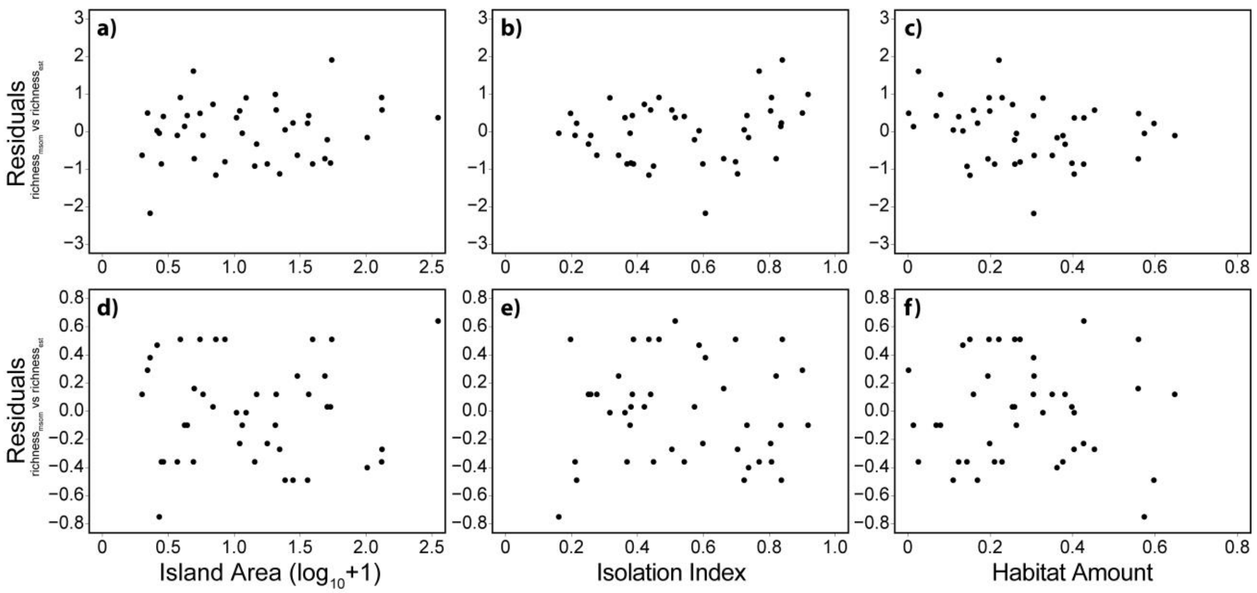

Figure S3. Residual plots showing the relationship between sampled vs. estimated species richness and island area, isolation, and habitat amount for beetles (a – c) and birds (d – f). Species richness was estimated using multi species occupancy models that accounted for species specific traits that affect detection probability (beetles – feeding guild, body size; birds – mass, max vocal frequency, and migration factor [migrant vs. resident], see Appendix S1: Table S2).

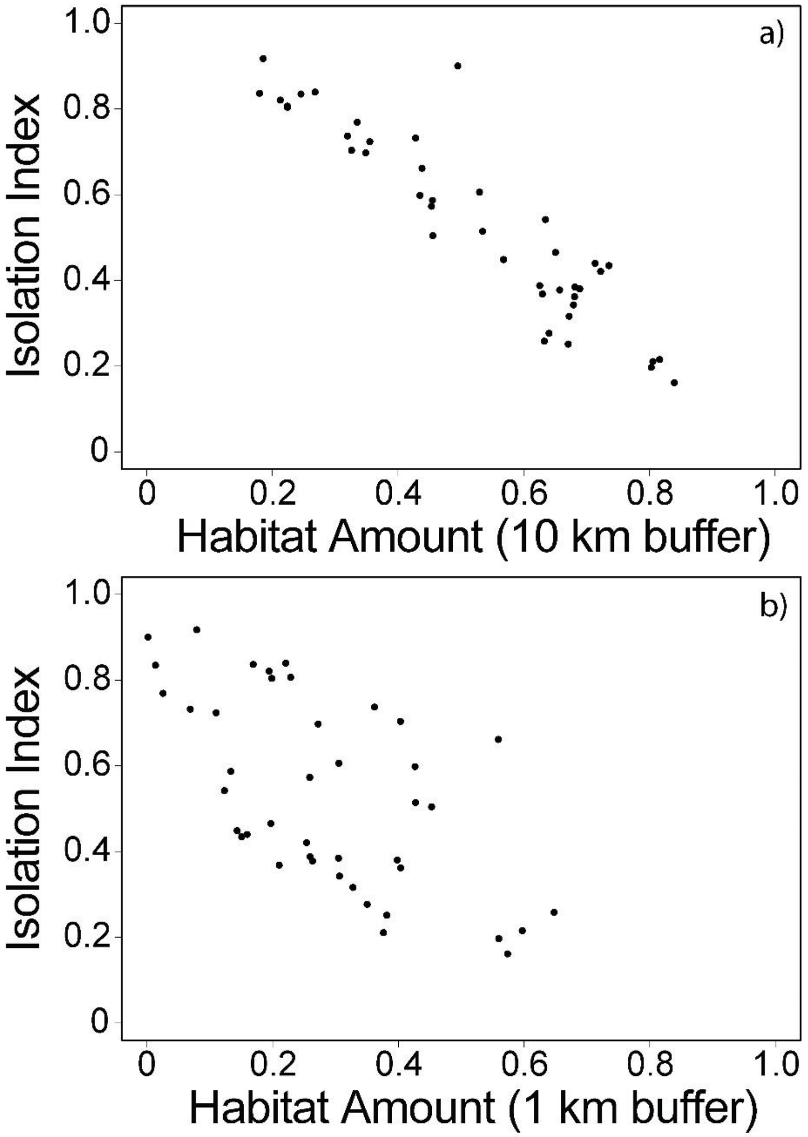

Figure S4. The relationship between isolation index and habitat amount at spatial extents of a) 10 km (r = -0.92, *P* = <0.001) and b) 1 km (r = -0.62 , *P* = <0.001).

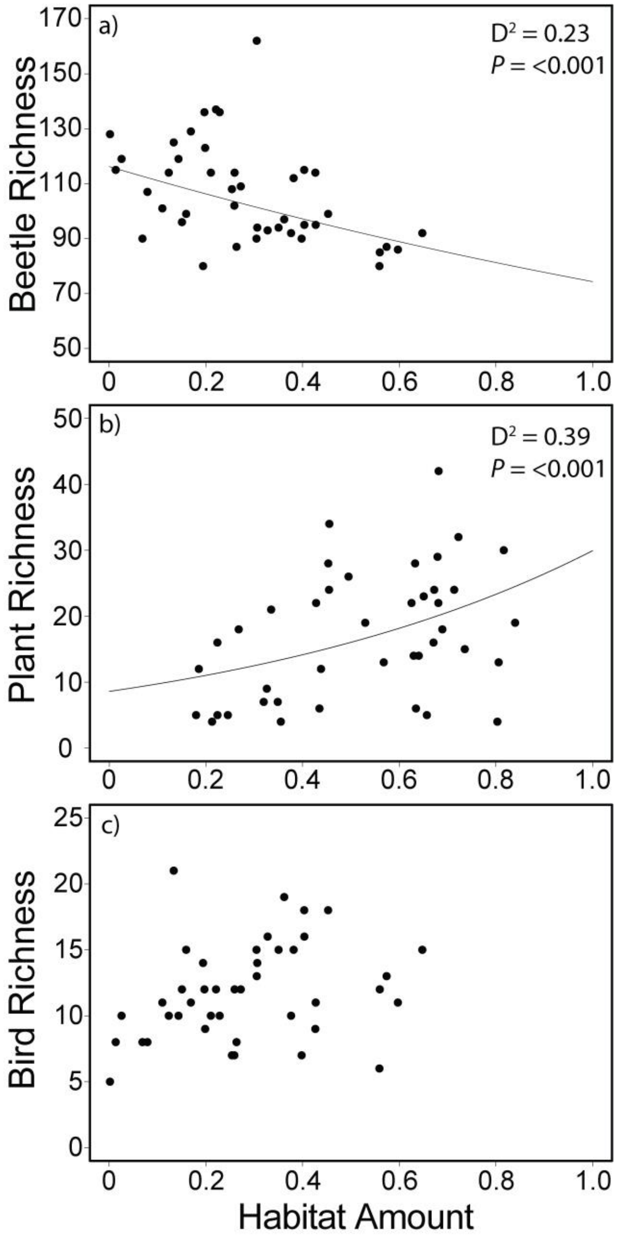

Figure S5. The relationship between species richness and habitat amount for beetles (a), plants (b), and birds (c). Habitat amount was determined at a spatial extent of 1 km for beetles and birds and 10 km for plants. Abbreviations: Deviance explained (D^2), p-value (*P*) of significant explanatory variables.

Literature Cited

Aalbu, R.L., Triplehorn, C.A., Campbell, J.M., Brown, K.W., Somerby, R.E., and D.B. Thomas. 2002. Tenebrionidae Latreille 1802. Pp 463-509. in Arnett, R.H., Thomas, M.C., Skelley, P.E., and J.H. Frank, editors. eds. American Beetles volume 2. CRC Press. Boca Raton, Florida.

Anderson, R.S. 1989. Revision of the subfamily Rhynchaeninae in North America (Coleoptera: Curculionidae). Transactions of the American Entomological Society 115: 207-312.

Arnett, R.H. Jr., and M.C. Thomas. 2000. American Beetles, Volume I: Archostemata, Myxophaga, Adephaga, Polyphaga: Staphyliniformia. CRC Press LLC, Boca Raton, FL. 464 pp. https://doi.org/10.1201/9781482274325

Arnett, R.H., Jr., Thomas, M.C., Skelley, P.E., J.H. Frank. 2002. American Beetles. Volume 2. Polyphaga: Scarabaeoidea through Curculionoidea. CRC Press LLC, Boca Raton, FL. 661 pp.

Assing, V., and P. Wunderle. 1995. A revision of the species of the subfamily Habrocerinae (Coleoptera: Staphylinidae) of the world. Revue Suisse de Zoologie 102: 307-359.

Baranowski, R. 1993. Revision of the genus *Leiodes* Latreille of North and Central America (Coleoptera: Leiodidae). Entomological Scandinavia Supplement 42: 1-149.

Barron, J.R. 1971. A revision of the Trogositidae of America north of Mexico (Coleoptera: Clerioidea). Memoirs of the Entomological Society of Canada 103: 1-143.

Barron, J.R. 1996. Review of Nearctic species of *Osotoma* (Coleoptera: Cleroidea, Trogositidae). Annals of the Entomological Society of America 89: 193-202.

Blake, D.H. 1943. The generic position of *Hypolampsis pilosa* (Illiger) and some related new species (Coleoptera: Halticidae). Proceedings of the Entomological Society of Washington 45: 207-221.

Bousquet, Y., and S. Laplante. 2006. The insects and arachnids of Canada. Part 24. Coleoptera Histeridae. NRC Research Press, Ottawa, 485 pp.

Bright, D.E. 1976. The insects and arachnids of Canada. Part 2. The bark beetles of Canada and Alaska (Coleoptera: Scolytidae). Agriculture Canada, Ottawa, 241 pp.

Casey, T. 1898. Studies in the Ptinidae, Cioidae, and Sphindidae of America. Journal of the New York Entomological Society 6: 61-93.

Dybas, H.S. 1966. Evidence for parthenogenesis in the featherwing beetles, with a taxonomic review of a new genus and eight new species (Coleoptera: Ptiliidae). Field Museum of Natural History 51: 11-52.

Gardiner, R.M., and D.A. Pollock. 2015. Revision of the Nearctic species of the genus *Ipthiminus* Spilman (Coleoptera: Tenebrionidae). ZooTaxa 4048: 352-391.

Goodrich, M.A., and C.A. Springer. 1999. The pleasing fungus beetles (Coleoptera: Erotylidae) of Nebraska. Transactions of the Nebraska Academy of Sciences 25: 53-71.

Hatch, M.H. 1933. Studies on the Leptodiridae (Catopidae) with descriptions of new species. Journal of the New York Entomological Society 41: 187-236.

Hatch, M.H. 1962. The beetles of the Pacific Northwest: Part 3: Pselaphidae and diversicornia I. Seattle: University of Washington Press.

Herman, L.E. 1975. Revision and phylogeny of the monogeneric subfamily Pseudopsinae for the world (Staphylinidae, Coleoptera). Bulletin of the American Museum of Natural History 155: 241-318.

Hinson, K.R., and R.J. Buss. 2015. New state records and identification of North American species of *Eurypogon* Motschulsky (Coleoptera: Artematopodidae), with a lectotype designation for *Eurypogon niger* (Melsheimer). The Coleopterists Bulletin 69: 768-772.

Horn, G.H. 1888. Miscellaneous Coleopterous studies. Transactions of the American Entomological Society and Proceedings of the Entomological Section of the Academy of Natural Sciences 15: 26-48.

Johnson, D., Kershaw, L.J., MacKinnon, A., and J. Pojar. 1995. Plants of the Western Boreal Forest and Aspen Parkland. Lone Pine Publishing. Edmonton, Alberta. 392 pp.

Johnson, P.J. 1991. Taxonomic notes, new records, and a key to the adults of North American Byrrhidae (Coleoptera). Proceedings of the Entomological Society of Washington 93: 322-332.

Johnston, M.A. and D.A. Pollock. 2025. Review of the Nearctic Salpinginae (Coleoptera: Salpingidae). The Coleopterists Bulletin 79: 111-124.

Kolibáč, J. 2013. Trogossitidae: a review of the beetle family, with a catalogue and keys. ZooKeys 366: 1-194.

Lawrence, J.F. 1971. Revision of the north American Ciidae (Coleoptera). Bulletin of the Museum of Comparative Zoology 142: 419-522.

Leavengood, J.M. 2008. The checkered beetles (Coleoptera: Cleridae) of Florida. M. Sc. thesis, University of Florida, United States.

Leighton, A.L. 2012. Flora of Saskatchewan, Fascicle 3, Sedges (Carex) of Saskatchewan. Nature Saskatchewan. Flora of Saskatchewan Association, Nature Saskatchewan, Regina, Saskatchewan.

Leighton, A.L., Harms V.L. 2014. Flora of Saskatchewan Fascicle 4, Grasses of Saskatchewan. Flora of Saskatchewan Association, Nature Saskatchewan, Regina, Saskatchewan.

LeSage, L. 1986. A taxonomic monograph of the Nearctic Galerucine genus *Ophraella* Wilcox (Coleoptera: Chrysomelidae). Memoirs of the Entomological Society of Canada 118: 3-75.

Lindroth, C.H. 1969. The ground-beetles of Canada and Alaska. Opuscula. Entomol. Suppl. 20, 24, 26, 29, 33, 34, 35: 1-1192.

Lopes-Andrade, C., Webster, R.P., Webster, V.L., Alderson, C.A., Hughes, C.C., and J.D. Sweeney. 2016. The Ciidae (Coleoptera) of New Brunswick, Canada: new records and synonyms. ZooKeys 573: 339-366.

Majka, C.G. 2010. Eucinetidae of the Maritime provinces of Canada. Journal of the Acadian Entomological Society 6: 16-21.

Majka, C.G. 2012. The Lampyridae of Atlantic Canada. Journal of the Acadian Entomological Society 8: 11-29.

Mank, E.W. 1939. Scotochroa and a closely allied new genus, *Scotochroides*, (Coleoptera, Melandryidae). The Canadian Entomologist 71: 181-183. doi: 10.4039/Ent71181-8

Parry, R.H. 1986. The systematics and biology of the flea beetle genus *Crepidodera* Chevrolat (Coleoptera: Chrysomelidae) in America north of Mexico. Insecta Mundi 1: 156-196.

Parsons, C.T. 1975. Revision of Nearctic Mycetophagidae (Coleoptera). The Coleopterists Bulletin 29: 93-108.

Peck, S.B., and M.C. Thomas. 1998. A distributional checklist of the Beetles (Coleoptera) of Florida._Arthropods of Florida and Neighboring Land Areas. Volume 16. Florida Department of Agriculture and Consumer Services, Gainesville. 180 pp.

Pelletier, G., Hébert C. 2014. The Cantharidae of eastern Canada and northeastern United States. Canadian Journal of Arthropod Identification 25: 1-246. doi:10.3752/cjai.2014.25

Pelletier, G., Hébert C. 2019. The Cryptophagidae of Canada and the northern United States of America. Canadian Journal of Arthropod Identification 40: 305 pp. doi:10.3752/cjai.2019.40

Pollock, D.A. 2002. Melandryidae Leach 1815. Pp 417-422. in Arnett, R.H., Thomas, M.C., Skelley, P.E., and J.H. Frank, editors. eds. American Beetles volume 2. CRC Press. Boca Raton, Florida.

Smetana, A. 1971. Revision of the tribe Quediini of America north of Mexico (Coleoptera: Staphylinidae). Memoirs of the Entomological Society of Canada 103: 1-303.

Smith, E.H. 1985. Revision of the genus *Phyllotreta* Chevrolat of America north of Mexico Part I. The maculate species (Coleoptera: Chrysomelidae, Alticinae). Fieldiana, Zoology 28: 1-168.

Warner, R.E. 1966. A review of *Hylobius* of North America, with a new species injurious to slash pine (Coleoptera: Curculionidae). The Coleopterists Bulletin 20: 65-81.

Wheeler, Q.D. 1979. Slime mold beetles of the genus *Anisotoma* (Leiodidae): classification and evolution. Systematic Entomology 4: 251-309.

White, R.E. 1966. Six new Anobiidae from North America with keys. Proceedings of the Entomological Society of Washington 68: 228-236.
